# Human filariasis—contributions of the *Litomosoides sigmodontis* and *Acanthocheilonema viteae* animal model

**DOI:** 10.1007/s00436-020-07026-2

**Published:** 2021-02-06

**Authors:** Frederic Risch, Manuel Ritter, Achim Hoerauf, Marc P. Hübner

**Affiliations:** 1 Institute for Medical Microbiology, Immunology and Parasitology (IMMIP), University Hospital Bonn, Bonn, Germany; 2 German Center for Infection Research (DZIF), partner site Bonn-Cologne, Bonn, Germany

**Keywords:** Lymphatic filariasis, Onchocerciasis, Rodent models, *Acanthocheilonema viteae*, *Litomosoides sigmodontis*, Drug development

## Abstract

Filariae are vector-borne parasitic nematodes that are endemic worldwide, in tropical and subtropical regions. Important human filariae spp. include *Onchocerca volvulus*, *Wuchereria bancrofti* and *Brugia* spp., and *Loa loa* and *Mansonella* spp. causing onchocerciasis (river blindness), lymphatic filariasis (lymphedema and hydrocele), loiasis (eye worm), and mansonelliasis, respectively. It is estimated that over 1 billion individuals live in endemic regions where filarial diseases are a public health concern contributing to significant disability adjusted life years (DALYs). Thus, efforts to control and eliminate filarial diseases were already launched by the WHO in the 1970s, especially against lymphatic filariasis and onchocerciasis, and are mainly based on mass drug administration (MDA) of microfilaricidal drugs (ivermectin, diethylcarbamazine, albendazole) to filarial endemic areas accompanied with vector control strategies with the goal to reduce the transmission. With the United Nations Sustainable Development Goals (SDGs), it was decided to eliminate transmission of onchocerciasis and stop lymphatic filariasis as a public health problem by 2030. It was also requested that novel drugs and treatment strategies be developed. Mouse models provide an important platform for anti-filarial drug research in a preclinical setting. This review presents an overview about the *Litomosoides sigmodontis* and *Acanthocheilonema viteae* filarial mouse models and their role in immunological research as well as preclinical studies about novel anti-filarial drugs and treatment strategies.

**Supplementary Information:**

The online version contains supplementary material available at 10.1007/s00436-020-07026-2.

## Human filarial species

Important human filariae spp. are *Onchocerca volvulus*, *Wuchereria bancrofti,* *Brugia spp*, *Loa loa* and *Mansonella spp*. Although the majority of infected individuals remain asymptomatic due to filarial-driven suppression of host immunity (Hoerauf and Brattig 2002; Adjobimey and Hoerauf 2010; Maizels et al. 2018; Ritter et al. 2018b, 2019; Alvar et al. 2020), filariae can influence disease outcome of concomitant infections and vaccination efficacy (Chatterjee et al. 2015; Santiago and Nutman 2016; Kroidl et al. 2016; Kabagenyi et al. 2020; Muhangi et al. 2007; Hillier et al. 2008; Elliott et al. 2010; Stensgaard et al. 2016; Mhimbira et al. 2017). Moreover, *O. volvulus*, *W. bancrofti*, and *Brugia* infections can lead to severe clinical symptoms and diseases in a subset of patients that develop strong inflammatory responses against the filariae. For example, the disease onchocerciasis caused by *Onchocerca volvulus* can lead to vision loss, blindness, and dermatitis including its severest form sowda (Adewole and Ayeni 2009; Edungbola et al. 1987; Katawa et al. 2015; Njim et al. 2015). Thus, onchocerciasis is a major public health problem (WHO Oncho 2020) and approximately 90 million people live at risk of contracting the disease worldwide, especially in Sub-Saharan Africa including 17 million infected and 270,000 permanently blind individuals (WHO 2018). Similarly, lymphatic filariasis (LF) caused by *Wuchereria bancrofti*, *Brugia timori* and *B. malayi* can lead to severe clinical symptoms including hydrocele, lymphedema, lymphangitis, and elephantiasis (WHO LF 2020; Rebollo and Bockarie 2017). It is estimated that 68 million LF patients with 19 million hydrocele and 17 million lymphedema cases exist worldwide (Ramaiah and Ottesen 2014). In addition, loiasis caused by *Loa loa*, also known as African eye worm, is characterized by distinct clinical manifestations like Calabar swelling, pruritis, arthralgia, and sporadic sub-conjunctival migration of the adult worms (Lukiana et al. 2006; Akue et al. 2011). Recent studies have highlighted both increased mortality and significant increases in DALYs associated with loiasis (Chesnais et al. 2017; Veletzky et al. 2020). In contrast to onchocerciasis and LF, loiasis is geographically restricted to forested areas in 11 Western and Central African countries (Zouré et al. 2011; Kelly-Hope et al. 2012) with approximately 13 million infected individuals (Fernandez-Soto et al. 2014). In contrast, despite mild clinical manifestations (subcutaneous swellings, skin rashes, and pleuritis), a distinct clinical symptom as shown by other filarial infections is missing in *Mansonella perstans*–, *M. streptocerca*–, or *M. ozzardi*–infected individuals (Simonsen et al. 2011; Hoerauf 2009; Asio et al. 2009a; Downes and Jacobsen 2010). Mansonelliasis is endemic in tropical parts of Latin America and large proportions of Sub-Saharan Africa. It is estimated that over 600 million people are at risk of infection in over 33 countries and 114 million people are infected with *M. perstans* (Simonsen et al. 2011; Kamtchum Tatuene et al. 2014). The absence of a specific clinical condition has resulted in a shortfall on mansonelliasis research. However, it was shown that *M. perstans* strongly modulates host immune responses (Ritter et al. 2018b) which might explain the increased susceptibility and worsened disease course of HIV, tuberculosis (TB), and malaria as well as lowered efficacy of bacillus Calmette-Guerin vaccination against TB in endemic regions (Muhangi et al. 2007; Hillier et al. 2008; Elliott et al. 2010; Stensgaard et al. 2016; Mhimbira et al. 2017).

Vector control and mass drug administration (MDA) programs like OCP (Onchocerciasis Control Programme), APOC (African programme for Onchocerciasis control), and OEPA (Onchocerciasis Elimination Program of the Americas) were implemented decades ago (WHO 2018; Tsalikis 1993), achieving the interruption of *O. volvulus* transmission in Cuba, Ecuador, Mexico, and Guatemala (Mauricio et al. 2017) and elimination in Mali and Senegal (Diawara et al. 2009; Traore et al. 2012). GPELF (Global Programme to Eliminate LF) prevented an estimated number of 96 million new LF cases over the last 13 years, and it is now estimated that infections dropped to 68 million LF patients and 19 million hydrocele and 17 million lymphedema cases (Ramaiah and Ottesen 2014; WHO 2015).

However, it is becoming obvious that onchocerciasis and LF cannot be eliminated from Africa in the near future solely depending on the microfilaricidal and temporally embryostatic drugs currently used for MDA (ivermectin with or without albendazole) (Dadzie et al. 2003; Basáñez et al. 2008; Churcher et al. 2009; Chandy et al. 2011; Rebollo and Bockarie 2017; Koudou et al. 2018; Babu and Kar 2004). Moreover, some *Loa loa*–infected individuals with high microfilariae numbers in the peripheral blood suffered from severe adverse events (SAEs) following intake of ivermectin or DEC during MDA programs (Chippaux et al. 1996; Gardon et al. 1997; Boussinesq et al. 1998; Padgett and Jacobsen 2008). This issue has compromised the elimination of onchocerciasis and LF in co-endemic areas (Boussinesq 2006; Padgett and Jacobsen 2008). Finally, regarding treatment for mansonelliasis, ivermectin has been shown to have little effect on *M. perstans* infection in Africa (Asio et al. 2009a, 2009b, 2009c; Wanji et al. 2016). Thus, further research on alternative treatment strategies and novel, macrofilaricidal drugs are urgently needed to achieve the goal of eliminating transmission of onchocerciasis and stop lymphatic filariasis as a public health problem by 2030. Interestingly, at the end of the 20th century, rickettsiae-like intracellular bacteria, called *Wolbachia*, which are present in human filarial nematodes except *Loa loa*, opened up novel possibilities for anti-filarial treatment strategies (reviewed in detail by Kozek and Rao 2007). Indeed, studies and clinical trials revealed that antibiotics such as doxycycline deplete *Wolbachia* from the adult filariae leading to permanent sterility and finally death of the adult filariae (macrofilaricidal activity) which is an advantage compared to microfilaricidal drugs such as ivermectin and diethylcarbamazine (Bandi et al. 1999; Hoerauf et al. 2000; Hoerauf et al. 2001, 2002, 2003a, 2003b).

Research about human filariae is limited due to the restricted access to human parasitic life stages. *In vitro* and *in vivo* models of human pathogenic filariae are urgently needed for the investigation of the parasite’s biology and immunomodulatory capacity as well as detection of novel treatment strategies and anti-filarial drugs. Thus, model organisms and rodent models of filariasis mimicking human filarial infections are mandatory for research and this review will focus on the *Litomosoides sigmodontis* and *Acanthocheilonema viteae* rodent models and their role in immunological research as well as preclinical studies on novel anti-filarial drugs and treatment strategies.

### Important aspects for the development of drugs for human filarial infections

Safety and efficacy are universal aspects for novel drugs that have to be evaluated before large scale human trials can be conducted. In the case of drugs intended for human filarial infections, there are three additional important properties that need to be addressed during preclinical development:

First, filariae have a complex life cycle during which the nematodes may pass through different organs and go through various stages of development. It is therefore challenging for a single drug to target all life cycle stages of any given filaria. As a result, drugs against filariae are commonly separated into *microfilaricidal* (ivermectin, diethylcarbamazine) and *macrofilaricidal* (flubendazole, oxfendazole, anti-*Wolbachia* compounds) drugs. Microfilaricidal drugs target the progeny of filarial worms (microfilariae), which are taken up by the vector to transmit the parasite, whereas macrofilaricidal drugs target the adult stage. A significant amount of research capacities is currently focused on the development and validation of novel macrofilaricidal treatment strategies (Bakowski and McNamara 2019; Jacobs et al. 2019; Taylor et al. 2019; Hübner et al. 2019a; Ehrens et al. 2020; Geary et al. 2019; Hübner et al. 2020; Hawryluk 2020). Since human pathogenic filariae can survive in the host for years (Geary and Mackenzie 2011), microfilaricidal strategies generally only block the transmission of infections temporarily and are unable to completely eliminate the parasite. MDA strategies based on macrofilaricidal drugs may be able to achieve elimination much quicker (Hawryluk 2020).

The second important property in the case of filaricidal drugs concerns the *speed* at which the filariae are cleared from the host. Rapid killing of the parasite may lead to a significant release of both worm antigen and *Wolbachia* which may trigger significant host immune responses and cause adverse reactions (reviewed in more detail in Geary and Mackenzie 2011; Budge et al. 2018). Two examples of this are as follows: (1) adverse reactions after MDA treatment against lymphatic filariasis are more common in microfilaremic than amicrofilaremic patients (Budge et al. 2018); (2) the aforementioned adverse reactions in *Loa loa*–infected patients with high microfilariae numbers after treatment with ivermectin (Chippaux et al. 1996; Gardon et al. 1997; Boussinesq et al. 1998; Padgett and Jacobsen 2008). As a result, it may be desirable for novel macrofilaricidal drugs to slowly eliminate worms over time (Geary and Mackenzie 2011). It has been shown that doxycycline, the prototype anti-wolbachial drug, shows this effect (Supali et al. 2008).

The third property concerns the question whether elimination of the parasite itself is necessary. Some filariae that infect humans cause only limited pathology (e.g., *Loa loa*, *Mansonella* spp.). In the case of onchocerciasis, the aetiological agents involved in blindness are the microfilariae and their *Wolbachia* endosymbionts (Saint André et al. 2002; Geary and Mackenzie 2011). *Permanent sterilization* is therefore a potentially acceptable alternative to macrofilaricidal activity. In addition, sterilization of worms that is achieved via elimination of *Wolbachia* may be used in conjunction with directly acting filaricidal drugs to lessen potential adverse reactions. Such a strategy, a combination of doxycycline (targeting *Wolbachia*) with melarsomine (macrofilaricide), is commonly used for the treatment against adult *Dirofilaria immitis* in dogs (American Heartworm Society 2014).

### Rodent models—a historical perspective

Rodents are among the most widely used model organisms in both basic and translational medical and biological research which includes filarial diseases. Model organisms like the nematodes *Acanthocheilonema viteae* (Krepkogorskaja 1933) and *Litomosoides sigmodontis* (Chandler 1931) have been studied for over 70 years and have contributed to major discoveries to the filarial research community.

In the following part of this review, we will focus on the history of biological and medical research using the filariae *Acanthocheilonema viteae* and *Litomosoides sigmodontis* in small rodents (mice, rats, hamsters). In addition, we will discuss how these two organisms have been instrumental in discovering and understanding the role of excretory-secretory products of helminths (Harnett et al. 1989; Haslam et al. 1999; Goodridge et al. 2005), improve and develop novel chemotherapies (Hewitt et al. 1947; Rao et al. 1990; Reddy et al. 1983; Hoerauf et al. 1999) as well as vaccines (Hartmann et al. 1997a; Lucius et al. 1991), and unravel the importance of the intracellular bacteria *Wolbachia* that is present in most human pathogenic filariae with the exception of *Loa loa* (McLaren et al. 1975; Vincent et al. 1975; Sironi et al. 1995; Hoerauf et al. 1999)*.*

### *Acanthocheilonema viteae* (Krepkogorskaja 1933)

*A. viteae* (formerly *Litosoma wite, L. witei, L. vitei, Dipetalonema vite, D. blanci, D. witeae, D. viteae*) was initially described in 1933 by Krepkogorskaja (Bain 1978). The natural hosts are the Libyan jird *Meriones libycus* and the great gerbil *Rhombomys opimus.* The parasite is transmitted via the tick *Ornithodorus tartakovskyi*. Under laboratory conditions, the parasite can also infect other rodents, such as the Mongolian jird *Meriones unguiculatus* (Johnson et al. 1974), the golden hamster *Mesocricetus auratus* (Pacheco 1970; Neilson and Forrester 1975), rats, the multimammate rat *Mastomys nataliensis* (Holdstock 1974; Sänger and Lämmler 1979), and to a limited extend mouse strains (Haque et al. 1980; Storey et al. 1989) as well as Shaw’s jird *Meriones shawi* (Lumb et al. 2020). *A. viteae* adult worms reside in the subcutaneous tissue and start releasing microfilariae (MF) 6–9 weeks postinfection. MF may be detectable for up to 15 months postinfection, while the adult worms themselves have a maximum lifespan of up to 2 years (Johnson et al. 1974; Worms et al. 1961).

The first experimental infections were described in 1953 by Baltazard et al. (the parasite was known as *Dipetalonema blanci* at the time) and in the following decade research focussed on describing the general biology, life cycle, and laboratory maintenance (Baltazard et al. 1953; Chabaud 1957; Worms et al. 1961; Terry et al. 1961). Following this fundamental work, investigations into host-parasite interactions, immune responses, and chemotherapeutic agents were carried out by numerous groups. Frank Hawking, the father of the famous Physicist Stephen Hawking, demonstrated the importance of the spleen in controlling the number of MF of *A. viteae* (and *L. sigmodontis, Dirofilaria immitis,* and *Dirofilaria repens*) (Hawking 1962) and postulated the proximate reason for the periodicity of MF in the peripheral blood based on MF movement and changes in oxygen tension and body temperature (Hawking and Clark 1967).

*A. viteae* was one of the first rodent filarial models used for preclinical drug testing, demonstrating (in parallel with *Brugia pahangi*) macrofilaricidal efficacy of flubendazole in jirds in the 1970s/1980s (Denham et al. 1979; Denham 1980; Court et al. 1988) and the recrudescence of MF in amicrofilaremic golden hamsters after treatment with immunosuppressive drugs (e.g., methyl prednisolone acetate and cyclophosphamide) (Neilson 1978). Subsequent studies in the *A. vit*ea*e Mastomys* model showed a predominant microfilaricidal efficacy of the macrocyclic lactones ivermectin and moxidectin (Zahner et al. 1987; Rao et al. 1987; Schares et al. 1994) and addressed the adverse reactions caused by diethylcarbamazine (DEC) (Singh et al. 1985).

**Fig. 1:**
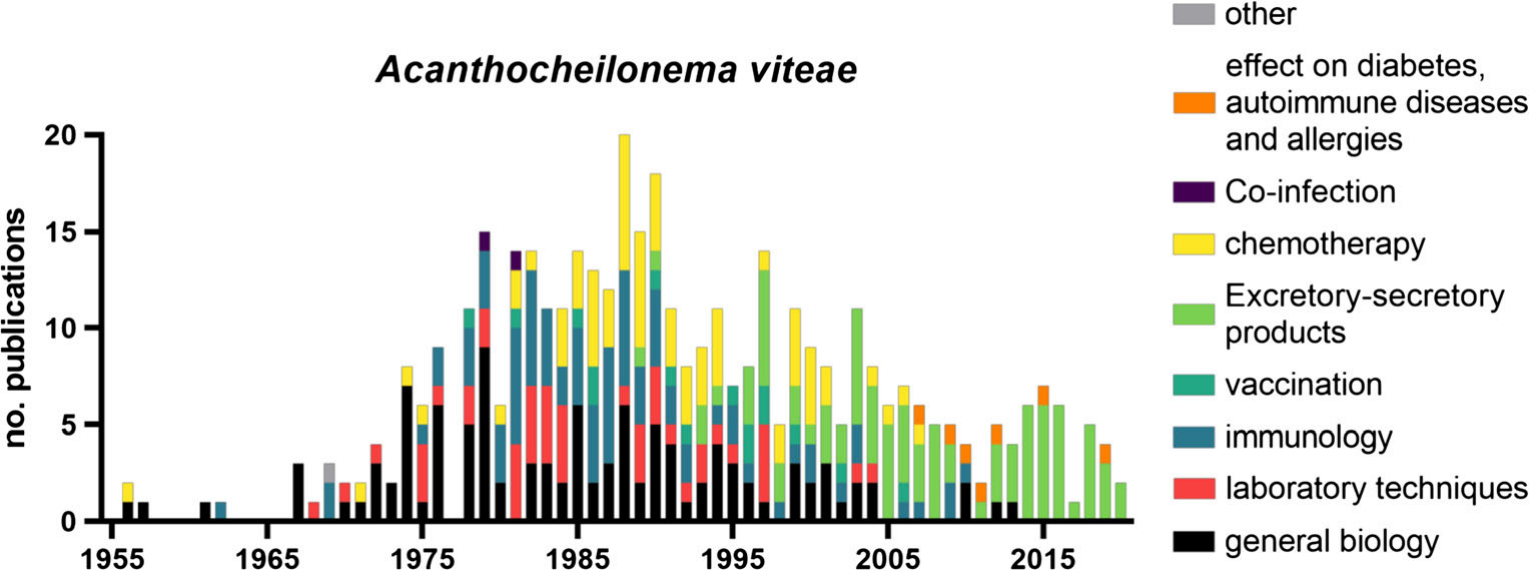
Publications on *Acanthocheilonema viteae* sorted by year and topic. List of publications was generated via PubMed search (see supplement table 1 for search parameters and complete list) and assigned one main topic each

While *A. viteae* is nowadays not regularly used for preclinical studies, the model became most famous by the discovery of two excretory-secretory (ES) immunomodulatory molecules (Fig. 1). With the discovery of Av17, a homologue to the human cystatin C (Hartmann et al. 1997a, 1997b), and ES-62 (Harnett et al. 1989), respectively, the area of filarial immunomodulation was introduced and this has led to the discovery of numerous other ES products (reviewed in detail in Maizels et al. 2018). The anti-inflammatory and immunomodulatory properties of excretory-secretory products such as ES-62 and cystatin and their potential application in treatments for autoimmune diseases such as arthritis and allergies as well as their use as therapeutics to influence the gut microbiome have been studied in great detail since their discovery and we refer the reader to the following publications for a more in-depth review on the topic (Harnett et al. 1999, Harnett and Harnett 2001a, 2001b, Harnett et al. 2004, Harnett and Harnett 2009; Al-Riyami and Harnett 2012; Pineda et al. 2014a, 2014b, 2015; Ebner et al. 2014; Rausch et al. 2018; Midha et al. 2018; Doonan et al. 2019; Langdon et al. 2019; Crowe et al. 2020).

In addition to the groundbreaking discovery of immunomodulatory excretory-secretory products, *A. viteae* has also been instrumental during the initial development of anti-*Wolbachia*-based treatment strategies for filarial diseases. *A. viteae*, unlike a number of human pathogenic filariae, such as the causative agents of lymphatic filariasis (*Wuchereria bancrofti, Brugia malayi* and *Brugia timori)* and onchocerciasis (*Onchocerca volvulus*) as well as the second rodent model to be covered in this review (*Litomosoides sigmodontis*), does not contain *Wolbachia*. As such, *A. viteae* has been used to show that the anti-filarial properties of certain antibiotics (tetracyclines like doxycycline and others) are indeed dependent on the presence of *Wolbachia*, since they do not affect *A. viteae* infection (Hoerauf et al. 1999)*.*

### *Litomosoides sigmodontis* (Chandler 1931)

*Litomosoides sigmodontis* (prior to 1989 conflated with *Litomosoides carinii* [Travassos 1919]) was described in 1931 as a filariae of the cotton rat *Sigmodon hispidus* and designated as the type species of the novel genus *Litomosoides* (Chandler 1931; Bain et al. 1989). Three years later, *L. sigmodontis* was erroneously synonymized with *L. carinii* (Travassos 1919) due to similarities in their morphology (Vaz 1934). Later studies by the group of Odile Bain established that *L. sigmodontis* and *L. carinii* are indeed two separate species and the names were corrected (Bain et al. 1989; Martin 2014).

Apart from the natural host *S. hispidus*, other experimental hosts include *Meriones unguiculatus* (Schneider et al. 1968), albino rats *Rattus norvegicus* (Ramakrishnan et al. 1961), *Mastomys nataliensis* (Pringle and King 1968), and mice *Mus musculus* (Hawking et al. 1947; Patra and Basu 1970; Petit et al. 1992). Mice in particular exhibit a variety of different responses to the infection with *L. sigmodontis* and display a more resistant or susceptible phenotype depending on the strain (Petit et al. 1992). BALB/c mice and to a lesser degree BALB/k and BALB/b mice were shown to be susceptible for infection with *L. sigmodontis* with a subset of animals developing microfilaremia (around 50%, 28%, and 6%, respectively), whereas CBA/HN, CBA/Ca, C3H/HeN, DBA/2N, B10, B10Br, and B10D2 do not develop microfilaremia, although all strains (in B10, B10Br, and B10D2 only in 28, 12, and 7% of mice) allowed the development into adult filariae (Petit et al. 1992). Therefore, the *L. sigmodontis* BALB/c mouse model mirrors the situation in humans infected with lymphatic filariasis where only a proportion of patients develops microfilaremia. This is one of the reasons why this mouse model has become widely used for filarial research.

*L. sigmodontis* is transmitted by the hematophagous mite *Ornithonyssus bacoti* which takes up MF from infected animals and transmits L3 larvae to (naive) animals during blood meals. The larvae then migrate through the skin and lymphatic system, enter the pulmonary blood circulation, and reach the pleural cavity within the first week postinfection (Karadjian et al. 2017; Kilarski et al. 2019). In susceptible mice (for example BALB/c mice), adult worms can be found in the pleural cavity after 28–30 days and MF are detectable in the peripheral blood after ~ 8 weeks (Hübner et al. 2009), whereas in semi-susceptible mice like C57BL/6 adult worms are cleared after 45 days and thus no MF are released (Petit et al. 1992). Therefore, the usage of different mouse strains allows the investigation of distinct research questions about parasite biology (e.g., latent vs patent infections), filarial-driven immune responses, associated morbidity, and treatment strategies.

The migration of L3 larvae through the pulmonary vessels and the presence of later worm stages in the pleural cavity makes the *L. sigmodontis* model ideal to study the pathogenesis of lung disease that can be associated with human pathogenic nematode infections (Vijayan 2007; Simonsen et al. 2011). Recent studies showed that the entry of L3s into the pleural cavity is associated with hemorrhages, granuloma formation, inflammation, and infiltration of neutrophils expressing calprotectin (Karadjian et al. 2017). The role of calprotectin, one of the most abundant proteins in neutrophils, during filarial infections is still unclear, but a follow-up study with calprotectin-deficient mice suggested an anti-inflammatory role of calprotectin that facilitates the migration of *L. sigmodontis* L3 larvae (Frohberger et al. 2020). Similarly, the *L. sigmodontis* rodent model has been used to investigate lung morbidity during chronic infections and different studies highlighted the role of the Th2 cytokines IL-4 and IL-5 in regulating inflammation at the site of infection (Ritter et al. 2017; Fercoq et al. 2019). The presence of MF in particular has a significant impact on inflammation in the lung and pleural cavity. Recent studies by Fercoq et al. (2020) demonstrated the formation of polyps on the pleura that consisted of up to 60% immune cells. The formation of these polyps which consisted mainly of CD3+ lymphocytes, CD68+ macrophages, and eosinophils correlated with the MF load (Fercoq et al. 2020).

Experimental studies on *Litomosoides sigmodontis* began in the 1940s with the discovery of the vector (Williams and Brown 1945) and the subsequent development of the required rearing techniques for the vector (Bertram et al. 1946; Scott et al. 1947; Hawking and Burroughs 1946). Similar to the work with *A. viteae*, the first studies in the following years focused on the basic biology of the parasite with investigations on the transmission by the vector (Freer 1953), life cycle (Williams 1948; Kershaw and Plackett 1948; Kershaw 1953), and identification of lymphatic vessels as the migratory pathway of infective larvae in the rodent host (Wenk 1967).

**Fig. 2:**
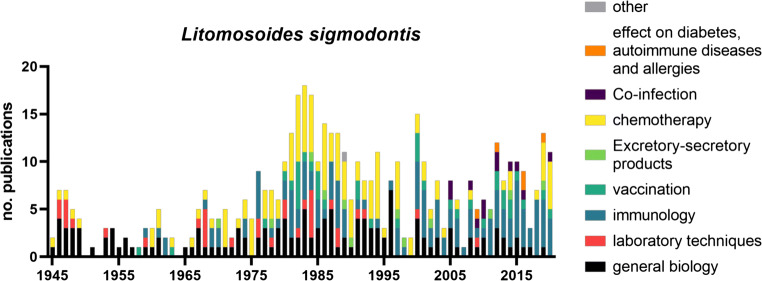
Publications on *Litomosoides sigmodontis* sorted by year and topic. List of publications was generated via PubMed search (see supplement table 1 for search parameters and complete list) and assigned one main topic each

By the mid to late 1950s, initial immunological studies in cotton rats were able to show a degree of immunity with slower worm growth, reduced molting, and fewer adult worms in animals with prior *L. sigmodontis* infections (Scott and Macdonald 1958) and that this protection persists for > 1 year even after the death of the worms from the primary infection (Macdonald and Scott 1958). Studies on the biology of the parasite, immune response to the infection, and development of new therapies have continued unabated in the following decades. Key results that helped to facilitate experimental studies include improvements to the laboratory maintenance and in particular generation of large numbers of L3 larvae via the “pelting method” (McCall 1976), dissection of mites (Nakamura et al. 1984), isolation of L3 larvae from the pleural cavity of Mongolian jirds 5 days after the infection (Hübner et al. 2009), and the validation of mice as an experimental host (Hawking and Burroughs 1946; Patra and Basu 1970; Petit et al. 1992). Mice have become the main experimental host for immunological research since the seminal paper by Petit et al. (1992) showed the susceptibility and maturation of *L. sigmodontis* in mice with a BALB/c background (Hoffmann et al. 2000; Finlay and Allen 2020). An overview about publications on *L. sigmodontis* is shown in Fig. 2.

*Key results in immunological research* include the discovery of a strong cross-reactivity between *Onchocerca volvulus* and *L. sigmodontis* antigen (Marcoullis and Gräsbeck 1976), the development of various vaccine protocols (primarily focused on attenuated L3 larvae with different adjuvants and treatment regiments) with varying degrees of success (reviewed in depth in Morris et al. 2013), IgE dependent killing of MF by neutrophils and macrophages (Mehta et al. 1980, 1982), and the significance of IL-4, IL-4R, IL-5, and IL-17 for the containment of the infection (Le Goff et al. 2000; Martin et al. 2000a, 2000b; Volkmann et al. 2001, 2003a; Taylor et al. 2006; Jenkins et al. 2011; Ritter et al. 2017, 2018a; Frohberger et al. 2019). Various studies have further shown that the protective immune responses against *L. sigmodontis* consist of both type 1 (Saeftel et al. 2003; Muhsin et al. 2018; Babayan et al. 2003) and type 2 immune responses. With regard to granulocytes, eosinophils were shown to mediate protection via the major basic protein, eosinophil peroxidase, eotaxin-1 (Specht et al. 2006; Gentil et al. 2014), and eosinophil extracellular DNA traps (EETosis; Ehrens et al. 2021), which contributes to MF and adult worm clearance (Martin et al. 2000a). Neutrophils on the other hand are required for protective responses to invading infective L3 larvae and adult worms (Al-Qaoud et al. 2000; Ajendra et al. 2016; Frohberger et al. 2020; Pionnier et al. 2016). Studies on basophils during *L. sigmodontis* infection showed that basophil responses are suppressed during chronic infection, and basophils have no effect on the adult worm burden during primary infection (Larson et al. 2012; Torrero et al. 2010; Hartmann et al. 2018). However, basophils amplify type 2 immune responses during primary *L. sigmodontis* infection and support vaccine responses using irradiated L3 larvae (Torrero et al. 2010, 2013).

Adaptive immune responses were also shown to be involved in protective immune responses against *L. sigmodontis*. Depletion of CD4+ T cells enhanced microfilaremia and *L. sigmodontis* adult worm recovery, while B cells and antibodies were shown to impact microfilaremia (Al-Qaoud et al. 1997; Martin et al. 2001). Studies with different B cell–deficient mouse strains have suggested a nuanced role of B cell subtypes in controlling microfilaremia as “μMT” (no mature B cells) and “B-less” mice (no immature or mature B cells) presented with equal or reduced MF numbers compared to BALB/c controls while B1 cell–deficient BALB Xid mice had an increased microfilariae load and increased adult worm burden (Al-Qaoud et al. 1998; Martin et al. 2001; Volkmann et al. 2001). The importance of T and B cells was further demonstrated in RAG2IL-2Rγ deficient mice which are on the semi-resistant C57BL/6 background, but lack B, T, and NK cells. These mice are highly susceptible to *L. sigmodontis* infection with 100% of mice developing microfilaremia (Layland et al. 2015).

BALB/c mice which develop a patent infection show a hyporesponsive phenotype compared to C57BL/6 mice (Finlay and Allen 2020). This difference in responsiveness appears to be strongly dependent on an expansion of regulatory T cells which suppresses the immune response to *L. sigmodontis* (Taylor et al. 2005, 2007, 2012). Furthermore, a distinct subset of dysfunctional Th2 cells occurs during the course of infection (Knipper et al. 2019, Taylor et. al. 2005; Finlay and Allen 2020). In addition to hyporesponsive CD4+ T cells (Knipper et al. 2019; Haben et al. 2013; van der Werf et al. 2013), cytotoxic T cell responses were also shown to be inhibited during *L. sigmodontis* infection (Kolbaum et al. 2012; Buerfent et al. 2015). CD4+ T cell hyporesponsiveness was shown to also be mediated by the expansion of alternatively activated macrophages via the release of the immunomodulatory mediator TGFβ (Taylor et al. 2006). These alternatively activated macrophages expand locally at the site of *L. sigmodontis* infection (Jenkins et al. 2011) and their discovery led to a series of groundbreaking studies concerning their role in tissue repair and immunoregulation (Taylor et al. 2006; Finlay and Allen 2020). Blocking the recruitment of monocyte-derived macrophages to the pleural cavity leads to an increase in T cell IL-4 production and reduced worm numbers in BALB/c mice suggesting a protective role of alternatively activated macrophages during *L. sigmodontis* infection (Campbell et al. 2018).

Similar to *A. viteae*, *L. sigmodontis* was shown to actively modulate the immune system of its host via cystatin (Pfaff et al. 2002). Furthermore, *L. sigmodontis* releases immunomodulatory small RNAs and exosomes (Buck et al. 2014; Quintana et al. 2019). This filarial immunomodulation was shown to impair vaccine responses (Haben et al. 2014; Hartmann et al. 2019), alter the course of co-infections (Hübner et al. 2012a; Gondorf et al. 2015; Dietze et al. 2016; Karadjian et al. 2014; Specht et al. 2010) and allergic sensitization (Dittrich et al. 2008), prevent the development of type 1 diabetes (Hübner et al. 2009, 2012b), and reduce diet-induced insulin resistance (Berbudi et al. 2016).

Moreover, the *L. sigmodontis* mouse model has been instrumental in understanding the role of *Wolbachia* beyond the mutualistic relationship with the parasite itself. For example, several studies showed that Toll-like and nucleotide-binding oligomerization domain (NOD)–like receptors and downstream signalling pathways are important for sensing *Wolbachia*, worm development, MF embryogenesis, and immunity against filariae (Pfarr et al. 2003; Brattig et al. 2004; Ajendra et al. 2016; Rodrigo et al. 2016; Wiszniewsky et al. 2019). For a more in-depth review of immune responses to *L. sigmodontis* in mice, we refer the reader to the recent review by Finlay and Allen (2020).

*Key results in the field of drug development with the Litomosoides model* include showing the microfilaricidal efficacy of diethylcarbamazine (DEC) in cotton rats (Hewitt et al. 1947), the lack of macrofilaricidal efficacy of DEC (Hawking et al. 1950), the loss of the microfilarial sheath following DEC treatment and co-localization of neutrophils and phagocytes with remaining MFs (Hawking et al. 1950; Schardein et al. 1968), the activity of ivermectin against developing stages (Campbell 1982), the suppression of microfilaremia by emodepside (Zahner et al. 2001), the macrofilaricidal activity of the benzimidazoles flubendazole (Zahner and Schares 1993; Hübner et al. 2019a) and oxfendazole (Hübner et al. 2020), and the development of treatment strategies against *Wolbachia* (Hoerauf et al. 1999; Specht et al. 2018).

These and other studies have been important in the preclinical development of the above-mentioned drugs. DEC was tested against infections with *W. bancrofti* in humans in the same year and a significant reduction in MF was observed (Santiago-Stevenson et al. 1947). First clinical trials for onchocerciasis in humans were started in 1981 and confirmed the microfilaricidal efficacy of ivermectin for up to 1 year after treatment (Aziz et al. 1982). Flubendazole, an inhibitor of tubulin polymerization, was also tested against *O. volvulus* but presented with two major issues (Dominguez-Vazquez et al. 1983; Geary et al. 2019). Firstly, flubendazole had very limited oral bioavailability and only achieved 100% macrofilaricidal activity in parenteral formulations. Secondly, flubendazole caused abscesses at the injection site during the original trial against *O. volvulus* in humans. Even though a formulation with high bioavailability after oral treatment was eventually found, the novel formulation has the risk to cause aneuploidia and was therefore not considered for further testing in humans (Lachau-Durand et al. 2019). Oxfendazole is one of the more recent macrofilaricidal candidates. Unlike flubendazole, oxfendazole has a high macrofilaricidal efficacy after oral administration against *L. sigmodontis* in BALB/c mice (Hübner et al. 2020). In addition, results from a phase 1 ascending dose study in humans showed that oxfendazole was well tolerated and caused no major side effects in relevant dosages (Bach et al. 2020).

As discussed earlier, rodent models have been instrumental in discovering the significance of *Wolbachia* in filariae and discovering antibiotics as novel therapeutic approaches and safe macrofilaricidal drugs for human pathogenic filariae. The current recommendations for doxycycline therapy for onchocerciasis and lymphatic filariasis are daily oral treatments for 4 to 6 weeks to achieve macrofilaricidal efficacy (Debrah et al. 2015; Klarmann et al. 2012; WHO 2019). Daily treatments with antibiotics for 4 to 6 weeks are not feasible for MDA and miss the target product profile (TPP) for novel (macrofilaricidal) drugs for onchocerciasis as defined by the *Drugs for Neglected Diseases initiative* (DNDi) and Bill & Melinda Gates Foundation*.* Therefore, *in vitro* screenings in *Wolbachia* insect cell lines and testing in animal models have been used to identify improved drugs (Clare et al. 2019; Bakowski and McNamara 2019). Studies with the *L. sigmodontis* rodent model identified and validated rifampicin (Volkmann et al. 2003b), ABBV-4083 (Taylor et al. 2019; Hübner et al. 2019b), corallopyronin A (Schiefer et al. 2012; Schiefer et al. 2020), and AWZ1066S (Hong et al. 2019) as promising novel anti-*Wolbachia* compounds (an overview of the current drugs and candidates and their mode of action is given in Table 1). Rifampicin belongs to the class of rifamycins and targets the bacterial DNA-dependent RNA-polymerase (McClure and Cech 1978). ABBV-4083 is an analogue of TylosinA which was designed to improve the bioavailability of TylosinA after oral treatment (von Geldern et al. 2019). Corallopyronin A was isolated from the myxobacterium *Corallococcus coralloides* and inhibits the bacterial DNA-dependent RNA-polymerase (Schaberle et al. 2015). AWZ1066S is an azaquinazoline that is highly specific and effective against *Wolbachia* (Hong et al. 2019).

**Table 1:** Overview of current and prospective anti-filarial compounds

Name	Mode of action	Status	Effect	Class	Clinical trials [ID]	Source
Doxycycline	Binds 30S ribosomal subunit, inhibition of protein synthesis	Used in individual therapy for onchocerciasis and lymphatic filariasis	*Wolbachia depletion/macrofilaricide*	Tetrazycline	ISRCTN65756724 [LF] ISRCTN14042737 [LF] ISRCTN06010453 [Oncho] ISRCTN68861628 [Oncho] ISRCTN71141922 [Oncho]	Klarman-Schulz et al. 2017 Taylor et al. 2010 Debrah et al. 2007 Debrah et al. 2015
Rifampicin	Binds DNA-dependent RNA-polymerase, inhibition of translation	Phase II clinical studies with the standard low dose have been performed. High dose clinical studies are scheduled.	*Wolbachia depletion/potential macrofilaricide*	Rifamycin	ISRCTN68861628 [Oncho] ISRCTN15216778 [LF]	Aljayyoussi et al. 2017
ABBV-4083	Binds 50S ribosomal subunit, inhibition of protein synthesis	Phase I clinical studies completed. Phase II clinical studies under preparation.	*Wolbachia depletion/potential macrofilaricide*	Macrolide		Taylor et al. 2019
AWZ-1066S	Inhibits protein synthesis	Under preparation for phase 1 clinical studies	*Wolbachia depletion/potential macrofilaricide*	Azaquinazoline		Hong et al. 2019
AN11251	Binds 50S ribosomal subunit, inhibition of protein synthesis	Backup clinical candidate	*Wolbachia depletion/potential macrofilaricide*	Pleuromutilin		Jacobs et al. 2019
Corallopyronin A	Inhibits DNA-dependent RNA-polymerase	under preparation for phase 1 clinical studies	*Wolbachia depletion/potential macrofilaricide*	α-Pyrone		Schiefer et al. 2012 Schiefer et al. 2020
Ivermectin (IVM)	Interferes with ligand-gated chloride channels	Used against onchocerciasis and lymphatic filariasis as mass drug administrations.	Microfilaricide	Avermectin	ISRCTN50035143 [Oncho]	Richard-Lenoble et al. 2003 Laing et al. 2017
Moxidectin	Exact mode of action is unknown	Potential alternative to ivermectin for mass drug administrations	Microfilaricide	Milbemycine	ISRCTN50035143 [Oncho]	Milton et al. 2020 Opoku et al. 2018
Diethylcarbamazine (DEC)	Alters metabolism of arachidonic acid	Used against lymphatic filariasis as part of mass drug administrations. Contraindicated in onchocerciasis patients	Microfilaricide	Piperazines	ISRCTN76875372 [Oncho]	Maizels and Denham 1992
Albendazole	Inhibits tubulin polymerization	Used against lymphatic filariasis as part of mass drug administrations; used in conjunction with diethylcarbamazine and/or ivermectin	May improve microfilaricidal efficacy of IVM/DEC	Benzimidazole	ISRCTN50035143 [Oncho] ISRCTN06010453 [Oncho] ISRCTN25831558 [Loiasis] ISRCTN56578422 [LF]	Macfarlane et al. 2019
Flubendazole	Inhibits tubulin polymerization	Currently not recommended for clinical use against human filarial diseases due to possible adverse events and limited bioavailability after oral treatment.	Potential macrofilaricide	Benzimidazole		Geary et al. 2019 Lachau-Durand et al. 2019
Oxfendazole	Inhibits tubulin polymerization	Phase I clinical studies completed.	Potential macrofilaricide	Benzimidazole		Hübner et al. 2020 Gonzalez et al. 2019
Emodepside	Interferes with potassium channel SLO-1	Phase I clinical studies completed. Phase II clinical studies under preparation.	Potential macrofilaricide	Cyclooctadepsipeptide		Kulke et al. 2017

Corallopyronin A and AWZ1066S are in preclinical development (with phase I studies in preparation) while ABBV-4083 has already completed phase I trials (Alami et al. 2019; von Geldern et al. 2019). In contrast, Rifampicin was already evaluated in a clinical study in Ghana in the early 2000s (Specht et al. 2008). The treatment failed to show a sufficient reduction in *Wolbachia* numbers, but this has been attributed to a suboptimal dose in humans (Aljayyoussi et al. 2017). Later studies with other rodent models, i.e., experimental infection of *M. unguiculatus* with *B. malayi* and implantation of *O. ochengi* in CB.17 SCID mice, showed that a shorter, high dose regimen has a higher speed of action than doxycycline, achieves > 90% *Wolbachia* reduction, and is not expected to lead to increased toxicity compared to low dose rifampicin (Aljayyoussi et al. 2017; Velásquez et al. 2018). As such, treatment with high dose rifampicin for < 2 weeks may be sufficient to achieve macrofilaricidal efficacy against the causative agents of lymphatic filariasis in humans (Ajjayyoussi et al. 2017).

### Current trends and developments

There have been a number of exciting developments in our field in the last decade. The search for novel treatment strategies has yielded a variety of drug candidates that may achieve safe, macrofilaricidal activity against human pathogenic filariae. ABBV-4083 (von Geldern et al. 2019; Taylor et al. 2019), emodepside (Kulke et al. 2017), and oxfendazole (Hübner et al. 2020; Bach et al. 2020) have already completed phase I clinical trials and phase II studies are in preparation (DNDi 2020). Corallopyronin A (Schiefer et al. 2020) and AWZ1066S (Hong et al. 2019) are in preclinical development and have so far shown promising results. Phase I studies for these candidates are in preparation. Although *in vitro* culture models for human filariae have been developed (Tippawangkosol et al. 2002; Falcone et al. 1995; Njouendou et al. 2017, 2018, 2019; Zofou et al. 2018; Fombad et al. 2019; Voronin et al. 2019), until now it is not possible to mimic the parasite biology in the human host and obtain all life stages, especially reproductive adult worms, *in vitro*. Therefore, *in vivo* models of filariasis are urgently needed for the investigation of the parasite’s biology, immunomodulatory capacity, and development of novel treatment strategies and anti-filarial drugs. Indeed, artificial human filarial infections in animal models, e.g., *B. malayi* in BALB/c SCID mice and *O. volvulus* in NSG mice (Duke 1957; Trees 1992; Lawrence et al. 1994; Patton et al. 2018; Halliday et al. 2014), have been established and recent studies showed that distinct mouse models, especially the RAG2IL-2Rγ-deficient mice, might be suitable to answer unresolved questions about human filarial infections and open up new avenues to develop, test, and validate novel treatment strategies against filariae (Fombad et al. 2019; Pionnier et al. 2019; Chunda et al. 2020). However, those results are limited since no complete life cycle of human filariae could be established *in vivo* until now and drug efficacy may be impaired in immunocompromised animals. Thus, further research on animal models is required that improves our testing of novel treatment strategies in preclinical studies and would allow the access of all parasite life stages, which is until now a critical issue also in regards to ethical concerns.

To summarize, rodent models for human filarial diseases have been studied for over 70 years and have been instrumental in uncovering basic biological properties of filariae, understanding immune responses to and immunomodulation by nematodes as well as developing novel therapies. While rodent models have been influential and successful, it is nonetheless important for researches to remember the limitations of model infections. Thus, additional validations with different model parasites or hosts are important factors in effective preclinical development.

## Supplementary information

ESM 1 (XLSX 90.1 kb)

## Data Availability

All data generated or analyzed during this study are included in this published article and its supplementary information files.

## References

[CR1] Adewole SO, Ayeni SK (2009). Clinical manifestation of onchocerciasis in Ise-Orun local Government, Ekiti State, Nigeria. Pak J Nutr.

[CR2] Adjobimey T, Hoerauf A (2010). Induction of immunoglobulin G4 in human filariasis: an indicator of immunoregulation. Ann Trop Med Parasitol.

[CR3] Ajendra J, Specht S, Ziewer S, Schiefer A, Pfarr K, Parčina M, Kufer TA, Hoerauf A, Hübner MP (2016). NOD2 dependent neutrophil recruitment is required for early protective immune responses against infectious Litomosoides sigmodontis L3 larvae. Sci Rep.

[CR4] Akue JP, Nkoghe D, Padilla C, Moussavou G, Moukana H, Mbou RA, Ollomo B, Leroy EM (2011). Epidemiology of concomitant infection due to Loa loa and Mansonella perstans in Gabon. PLoS Negl Trop Dis.

[CR5] Alami NN, Carter DC, Kwatra NV, Zhao W, Snodgrass L, Porcalla AR, Klein CE, Cohen DE, Gallenberg LA, Carr RA, Marsh KC (2019) Anti-Wolbachia Candidate ABBV-4083: Phase 1 safety and pharmacokinetics clinical trial in healthy adults. Am J Trop Med Hyg 101:392-392

[CR6] Aljayyoussi G, Tyrer HE, Ford L, Sjoberg H, Pionnier N, Waterhouse D, Davies J, Gamble J, Metuge H, Cook DAN, Steven A, Sharma R, Guimaraes AF, Clare RH, Cassidy A, Johnston KL, Myhill L, Hayward L, Wanji S, Turner JD, Taylor MJ, Ward SA (2017). Short-course, high-dose rifampicin achieves Wolbachia depletion predictive of curative outcomes in preclinical models of lymphatic filariasis and onchocerciasis. Sci Rep.

[CR7] Al-Qaoud KM, Taubert A, Zahner H, Fleischer B, Hoerauf A (1997). Infection of BALB/c mice with the filarial nematode Litomosoides sigmodontis: role of CD4+ T cells in controlling larval development. Infect Immun.

[CR8] Al-Qaoud KM, Fleischer B, Hoerauf A (1998). The Xid defect imparts susceptibility to experimental murine filariosis--association with a lack of antibody and IL-10 production by B cells in response to phosphorylcholine. Int Immunol.

[CR9] Al-Qaoud KM, Pearlman E, Hartung T, Klukowski J, Fleischer B, Hoerauf A (2000). A new mechanism for IL-5-dependent helminth control: neutrophil accumulation and neutrophil-mediated worm encapsulation in murine filariasis are abolished in the absence of IL-5. Int Immunol.

[CR10] Al-Riyami L, Harnett W (2012). Immunomodulatory properties of ES-62, a phosphorylcholine-containing glycoprotein secreted by Acanthocheilonema viteae. Endocr Metab Immune Disord Drug Targets.

[CR11] Alvar J, Alves F, Bucheton B, Burrows L, Büscher P, Carrillo E, Felger I, Hübner MP, Moreno J, Pinazo MJ, Ribeiro I, Sosa-Estani S, Specht S, Tarral A, Wourgaft NS, Bilbe G (2020). Implications of asymptomatic infection for the natural history of selected parasitic tropical diseases. Semin Immunopathol.

[CR12] American Heartworm Society (2014) Summary of the current canine guidelines for the prevention, diagnosis, and management of heartworm (Dirofilaria immitis) infection in dogs. https://www.heartwormsociety.org/images/pdf/Canine-Guidelines-Summary.pdf

[CR13] Asio SM, Simonsen PE, Onapa AW (2009). Mansonella perstans filariasis in Uganda: patterns of microfilaraemia and clinical manifestations in two endemic communities. Trans R Soc Trop Med Hyg.

[CR14] Asio SM, Simonsen PE, Onapa AW (2009). Mansonella perstans: safety and efficacy of ivermectin alone, albendazole alone and the two drugs in combination. Ann Trop Med Parasitol.

[CR15] Asio SM, Simonsen PE, Onapa AW (2009). A randomised, double-blind field trial of ivermectin alone and in combination with albendazole for the treatment of Mansonella perstans infections in Uganda. Trans R Soc Trop Med Hyg.

[CR16] Aziz MA, Diallo S, Diop IM, Lariviere M, Porta M (1982). Efficacy and tolerance of ivermectin in human onchocerciasis. Lancet..

[CR17] Babayan S, Ungeheuer MN, Martin C, Attout T, Belnoue E, Snounou G, Rénia L, Korenaga M, Bain O (2003). Resistance and susceptibility to filarial infection with Litomosoides sigmodontis are associated with early differences in parasite development and in localized immune reactions. Infect Immun.

[CR18] Babu BV, Kar SK (2004). Coverage, compliance and some operational issues of mass drug administration during the programme to eliminate lymphatic filariasis in Orissa, India. Trop Med Int Health.

[CR19] Bach T, Galbiati S, Kennedy JK, Deye G, Nomicos EYH, Codd EE, Garcia HH, Horton J, Gilman RH, Gonzalez AE, Winokur P, An G (2020). Pharmacokinetics, safety, and tolerability of oxfendazole in healthy adults in an open-label phase 1 multiple ascending dose and food effect study. Antimicrob Agents Chemother.

[CR20] Bain O (1978) Litosoma wite Krepkogorskaya 1933 (nematoda); proposed correction to Litosoma viteae. Bull Zool Nomencl 35.part 1

[CR21] Bain O, Petit G, Diagne M (1989). Etude de quelques Litomosoides parasites de rongeurs; consequences t taxonomiques [Litomosoides, parasites of rodents; taxonomic consequences]. Ann Parasitol Hum Comp.

[CR22] Bakowski MA, McNamara CW (2019). Advances in antiwolbachial drug discovery for treatment of parasitic filarial worm infections. Trop Med Infect Dis.

[CR23] Baltazard M, Chabaud AG, Mofidi C, Minou A (1953). Une nouvelle filaire de laboratoire [A new laboratory filaria]. Ann Parasitol Hum Comp.

[CR24] Bandi C, McCall JW, Genchi C, Corona S, Venco L, Sacchi L (1999). Effects of tetracycline on the filarial worms Brugia pahangi and Dirofilaria immitis and their bacterial endosymbionts Wolbachia. Int J Parasitol.

[CR25] Basáñez MG, Pion SD, Boakes E, Filipe JA, Churcher TS, Boussinesq M (2008). Effect of single-dose ivermectin on Onchocerca volvulus: a systematic review and meta-analysis. Lancet Infect Dis.

[CR26] Berbudi A, Surendar J, Ajendra J, Gondorf F, Schmidt D, Neumann AL, Wardani AP, Layland LE, Hoffmann LS, Pfeifer A, Hoerauf A, Hübner MP (2016). Filarial infection or antigen administration improves glucose tolerance in diet-induced obese mice. J Innate Immun.

[CR27] Bertram DS, Unsworth K, Gordon RM (1946). The biology and maintenance of Liponyssus bacoti Hirst, 1913, and an investigation into its role as a vector of Litomosoides carinii to cotton rats and white rats, together with some observations on the infection in the white rats. Ann Trop Med Parasitol.

[CR28] Boussinesq M (2006). Loiasis. Ann Trop Med Parasitol.

[CR29] Boussinesq M, Gardon J, Gardon-Wendel N, Kamgno J, Ngoumou P, Chippaux JP (1998). Three probable cases of Loa loa encephalopathy following ivermectin treatment for onchocerciasis. Am J Trop Med Hyg.

[CR30] Brattig NW, Bazzocchi C, Kirschning CJ, Reiling N, Büttner DW, Ceciliani F, Geisinger F, Hochrein H, Ernst M, Wagner H, Bandi C, Hoerauf A (2004). The major surface protein of Wolbachia endosymbionts in filarial nematodes elicits immune responses through TLR2 and TLR4. J Immunol.

[CR31] Buck AH, Coakley G, Simbari F, McSorley HJ, Quintana JF, Le Bihan T, Kumar S, Abreu-Goodger C, Lear M, Harcus Y, Ceroni A, Babayan SA, Blaxter M, Ivens A, Maizels RM (2014). Exosomes secreted by nematode parasites transfer small RNAs to mammalian cells and modulate innate immunity. Nat Commun.

[CR32] Budge PJ, Herbert C, Andersen BJ, Weil GJ (2018). Adverse events following single dose treatment of lymphatic filariasis: observations from a review of the literature. PLoS Negl Trop Dis.

[CR33] Buerfent BC, Gondorf F, Wohlleber D, Schumak B, Hoerauf A, Hübner MP (2015). Escherichia coli-induced immune paralysis is not exacerbated during chronic filarial infection. Immunology.

[CR34] Campbell WC (1982). Efficacy of the avermectins against filarial parasites: a short review. Vet Res Commun.

[CR35] Campbell SM, Knipper JA, Ruckerl D, Finlay CM, Logan N, Minutti CM, Mack M, Jenkins SJ, Taylor MD, Allen JE (2018). Myeloid cell recruitment versus local proliferation differentiates susceptibility from resistance to filarial infection. Elife..

[CR36] Chabaud AG (1957). Synonymie de Dipetalonema blanci et de Litomosa vite [Synonymy of Dipetalonema blanci and Litomosa fast]. Ann Parasitol Hum Comp.

[CR37] Chandler, A. C. (1931). New genera and species of nematode worms. Proc US Natl Museum.

[CR38] Chandy A, Thakur AS, Singh MP, Manigauha A (2011). A review of neglected tropical diseases: filariasis. Asian Pac J Trop Med.

[CR39] Chatterjee S, Clark CE, Lugli E, Roederer M, Nutman TB (2015). Filarial infection modulates the immune response to Mycobacterium tuberculosis through expansion of CD4+ IL-4 memory T cells. J Immunol.

[CR40] Chesnais CB, Takougang I, Paguélé M, Pion SD, Boussinesq M (2017). Excess mortality associated with loiasis: a retrospective population-based cohort study. Lancet Infect Dis.

[CR41] Chippaux JP, Boussinesq M, Gardon J, Gardon-Wendel N, Ernould JC (1996). Severe adverse reaction risks during mass treatment with ivermectin in loiasis-endemic areas. Parasitol Today.

[CR42] Chunda VC, Ritter M, Bate A, Gandjui NVT, Esum ME, Fombad FF, Njouendou AJ, Ndongmo PWC, Taylor MJ, Hoerauf A, Layland LE, Turner JD, Wanji S (2020). Comparison of immune responses to Loa loa stage-specific antigen extracts in Loa loa-exposed BALB/c mice upon clearance of infection. Parasit Vectors.

[CR43] Churcher TS, Pion SD, Osei-Atweneboana MY, Prichard RK, Awadzi K, Boussinesq M, Collins RC, Whitworth JA, Basáñez MG (2009). Identifying sub-optimal responses to ivermectin in the treatment of River Blindness. Proc Natl Acad Sci U S A.

[CR44] Clare RH, Bardelle C, Harper P, Hong WD, Börjesson U, Johnston KL, Collier M, Myhill L, Cassidy A, Plant D, Plant H, Clark R, Cook DAN, Steven A, Archer J, McGillan P, Charoensutthivarakul S, Bibby J, Sharma R, Nixon GL, Slatko BE, Cantin L, Wu B, Turner J, Ford L, Rich K, Wigglesworth M, Berry NG, O'Neill PM, Taylor MJ, Ward SA (2019). Industrial scale high-throughput screening delivers multiple fast acting macrofilaricides. Nat Commun.

[CR45] Court JP, Stables JN, Lees GM, Martin-Short MR, Rankin R (1988). Dipetalonema viteae and Brugia pahangi transplant infections in gerbils for use in antifilarial screening. J Helminthol.

[CR46] Crowe J, Lumb FE, Doonan J, Broussard M, Tarafdar A, Pineda MA, Landabaso C, Mulvey L, Hoskisson PA, Babayan SA, Selman C, Harnett W, Harnett MM (2020). The parasitic worm product ES-62 promotes health- and life-span in a high calorie diet-accelerated mouse model of ageing. PLoS Pathog.

[CR47] Dadzie Y, Neira M, Hopkins D (2003). Final report of the conference on the eradicability of onchocerciasis. Filaria J.

[CR48] Debrah AY, Mand S, Marfo-Debrekyei Y, Batsa L, Pfarr K, Buttner M, Adjei O, Buttner D, Hoerauf A (2007). Macrofilaricidal effect of 4 weeks of treatment with doxycycline on Wuchereria bancrofti. Tropical Med Int Health.

[CR49] Debrah AY, Specht S, Klarmann-Schulz U, Batsa L, Mand S, Marfo-Debrekyei Y, Fimmers R, Dubben B, Kwarteng A, Osei-Atweneboana M, Boakye D, Ricchiuto A, Büttner M, Adjei O, Mackenzie CD, Hoerauf A (2015). Doxycycline leads to sterility and enhanced killing of female Onchocerca volvulus worms in an area with persistent microfilaridermia after repeated ivermectin treatment: a randomized, placebo-controlled, double-blind trial. Clin Infect Dis.

[CR50] Denham DA (1980). Anthelmintic properties of flubendazole against Dipetalonema viteae in jirds. Trans R Soc Trop Med Hyg.

[CR51] Denham DA, Samad R, Cho SY, Suswillo RR, Skippins SC (1979). The anthelmintic effects of flubendazole on Brugia pahangi. Trans R Soc Trop Med Hyg.

[CR52] Diawara L, Traoré MO, Badji A, Bissan Y, Doumbia K, Goita SF, Konaté L, Mounkoro K, Sarr MD, Seck AF, Toé L, Tourée S, Remme JHF (2009) Feasibility of Onchocerciasis Elimination with Ivermectin Treatment in Endemic Foci in Africa: First Evidence from Studies in Mali and Senegal. PLoS Negl Trop Dis 3(7):e497 10.1371/journal.pntd.0000497 PMC2710500 19621091

[CR53] Dietze KK, Dittmer U, Koudaimi DK, Schimmer S, Reitz M, Breloer M, Hartmann W (2016). Filariae-retrovirus co-infection in mice is associated with suppressed virus-specific IgG immune response and higher viral loads. PLoS Negl Trop Dis.

[CR54] Dittrich AM, Erbacher A, Specht S, Diesner F, Krokowski M, Avagyan A, Stock P, Ahrens B, Hoffmann WH, Hoerauf A, Hamelmann E (2008). Helminth infection with Litomosoides sigmodontis induces regulatory T cells and inhibits allergic sensitization, airway inflammation, and hyperreactivity in a murine asthma model. J Immunol.

[CR55] Dominguez-Vazquez A, Taylor HR, Greene BM, Ruvalcaba-Macias AM, Rivas-Alcala AR, Murphy RP, Beltran-Hernandez F (1983). Comparison of flubendazole and diethylcarbamazine in treatment of onchocerciasis. Lancet..

[CR56] Doonan J, Tarafdar A, Pineda MA, Lumb FE, Crowe J, Khan AM, Hoskisson PA, Harnett MM, Harnett W (2019). The parasitic worm product ES-62 normalises the gut microbiota bone marrow axis in inflammatory arthritis. Nat Commun.

[CR57] Downes BL, Jacobsen KH (2010). A systematic review of the epidemiology of mansonelliasis. Afr J Infect Dis.

[CR58] Duke BO (1957). Experimental transmission of Loa loa from man to monkey. Nature..

[CR59] Ebner F, Hepworth MR, Rausch S, Janek K, Niewienda A, Kühl A, Henklein P, Lucius R, Hamelmann E, Hartmann S (2014). Therapeutic potential of larval excretory/secretory proteins of the pig whipworm Trichuris suis in allergic disease. Allergy..

[CR60] Edungbola LD, Watts SJ, Kayode OO (1987). Endemicity and striking manifestations of onchocerciasis in Shao, Kwara State. Afr J Med Med Sci.

[CR61] Ehrens A, Lenz B, Neumann AL, Giarrizzo S, Reichwald JJ, Frohberger SJ, Stamminger W, Buerfent BC, Fercoq F, Martin C, Kulke D, Hoerauf A, Hübner MP (2021) Microfilariae Trigger Eosinophil Extracellular DNA Traps in a Dectin-1-Dependent Manner. Cell Reports 34(2):108621 10.1016/j.celrep.2020.108621 33440150

[CR62] Ehrens A, Lunde CS, Jacobs RT, Struever D, Koschel M, Frohberger SJ, Lenz F, Fendler M, Turner JD, Ward SA, Taylor MJ, Freund YR, Stefanakis R, Easom E, Li X, Plattner JJ, Hoerauf A, Hübner MP (2020). In vivo efficacy of the boron-pleuromutilin AN11251 against Wolbachia of the rodent filarial nematode Litomosoides sigmodontis. PLoS Negl Trop Dis.

[CR63] Elliott AM, Mawa PA, Webb EL, Nampijja M, Lyadda N, Bukusuba J, Kizza M, Namujju PB, Nabulime J, Ndibazza J, Muwanga M, Whitworth JA (2010). Effects of maternal and infant co-infections, and of maternal immunisation, on the infant response to BCG and tetanus immunisation. Vaccine.

[CR64] Falcone FH, Zahner H, Schlaak M, Haas H (1995). In vitro cultivation of third-stage larvae of Brugia malayi to the young adult stage. Trop Med Parasitol.

[CR65] Fercoq F, Remion E, Frohberger SJ, Vallarino-Lhermitte N, Hoerauf A, Le Quesne J, Landmann F, Hübner MP, Carlin LM, Martin C (2019). IL-4 receptor dependent expansion of lung CD169+ macrophages in microfilaria-driven inflammation. PLoS Negl Trop Dis.

[CR66] Fercoq F, Remion E, Vallarino-Lhermitte N, Alonso J, Raveendran L, Nixon C, Le Quesne J, Carlin LM, Martin C (2020). Microfilaria-dependent thoracic pathology associated with eosinophilic and fibrotic polyps in filaria-infected rodents. Parasit Vectors.

[CR67] Fernandez-Soto P, Mvoulouga PO, Akue JP, Aban JL, Santiago BV, Sanchez MC, Muro A (2014). Development of a highly sensitive loop-mediated isothermal amplification (LAMP) method for the detection of Loa loa. PLoS One.

[CR68] Finlay CM, Allen JE (2020). The immune response of inbred laboratory mice to Litomosoides sigmodontis: a route to discovery in myeloid cell biology. Parasite Immunol.

[CR69] Fombad FF, Njouendou AJ, Ndongmo PC, Ritter M, Chunda VC, Metuge HM, Gandjui NVT, Enyong P, Njiokou F, Hoerauf A, Mackenzie CD, Wanji S (2019). Effect of flubendazole on developing stages of Loa loa in vitro and in vivo: a new approach for screening filaricidal agents. Parasit Vectors.

[CR70] Freer PM (1953). Observations on the early fate of the microfilariae of Litomosoides carinii (Travassos, 1919), filarial parasite of the cotton rat, after their ingestion by the vector, Bdellonyssus bacoti (Hirst, 1913). Ann Trop Med Parasitol.

[CR71] Frohberger SJ, Ajendra J, Surendar J, Stamminger W, Ehrens A, Buerfent BC, Gentil K, Hoerauf A, Hübner MP (2019). Susceptibility to L. sigmodontis infection is highest in animals lacking IL-4R/IL-5 compared to single knockouts of IL-4R, IL-5 or eosinophils. Parasit Vectors.

[CR72] Frohberger SJ, Fercoq F, Neumann AL, Surendar J, Stamminger W, Ehrens A, Karunakaran I, Remion E, Vogl T, Hoerauf A, Martin C, Hübner MP (2020). S100A8/S100A9 deficiency increases neutrophil activation and protective immune responses against invading infective L3 larvae of the filarial nematode Litomosoides sigmodontis. PLoS Negl Trop Dis.

[CR73] Gardon J, Gardon-Wendel N, Demanga N, Kamgno J, Chippaux JP, Boussinesq M (1997). Serious reactions after mass treatment of onchocerciasis with ivermectin in an area endemic for Loa loa infection. Lancet.

[CR74] Geary TG, Mackenzie CD (2011). Progress and challenges in the discovery of macrofilaricidal drugs. Expert Rev Anti-Infect Ther.

[CR75] Geary TG, Mackenzie CD, Silber SA (2019). Flubendazole as a macrofilaricide: history and background. PLoS Negl Trop Dis.

[CR76] Gentil K, Lentz CS, Rai R, Muhsin M, Kamath AD, Mutluer O, Specht S, Hübner MP, Hoerauf A (2014). Eotaxin-1 is involved in parasite clearance during chronic filarial infection. Parasite Immunol.

[CR77] Gondorf F, Berbudi A, Buerfent BC, Ajendra J, Bloemker D, Specht S, Schmidt D, Neumann AL, Layland LE, Hoerauf A, Hübner MP (2015). Chronic filarial infection provides protection against bacterial sepsis by functionally reprogramming macrophages. PLoS Pathog.

[CR78] Gonzalez AE, Codd EE, Horton J, Garcia HH, Gilman RH (2019). Oxfendazole: a promising agent for the treatment and control of helminth infections in humans. Expert Rev Anti-Infect Ther.

[CR79] Goodridge HS, Marshall FA, Else KJ, Houston KM, Egan C, Al-Riyami L, Liew FY, Harnett W, Harnett MM (2005). Immunomodulation via novel use of TLR4 by the filarial nematode phosphorylcholine-containing secreted product, ES-62. J Immunol.

[CR80] Haben I, Hartmann W, Specht S, Hoerauf A, Roers A, Müller W, Breloer M (2013). T-cell-derived, but not B-cell-derived, IL-10 suppresses antigen-specific T-cell responses in Litomosoides sigmodontis-infected mice. Eur J Immunol.

[CR81] Haben I, Hartmann W, Breloer M (2014). Nematode-induced interference with vaccination efficacy targets follicular T helper cell induction and is preserved after termination of infection. PLoS Negl Trop Dis.

[CR82] Halliday A, Guimaraes AF, Tyrer HE, Metuge HM, Patrick CN, Arnaud KO, Kwenti TD, Forsbrook G, Steven A, Cook D, Enyong P, Wanji S, Taylor MJ, Turner JD (2014). A murine macrofilaricide pre-clinical screening model for onchocerciasis and lymphatic filariasis. Parasit Vectors.

[CR83] Haque A, Worms MJ, Ogilvie BM, Capron A (1980). Dipetalonema viteae: microfilariae production in various mouse strains and in nude mice. Exp Parasitol.

[CR84] Harnett MM, Harnett W (2001). Antigen receptor signaling is subverted by an immunomodulatory product secreted by a filarial nematode. Arch Immunol Ther Exp.

[CR85] Harnett W, Harnett MM (2001). Modulation of the host immune system by phosphorylcholine-containing glycoproteins secreted by parasitic filarial nematodes. Biochim Biophys Acta.

[CR86] Harnett W, Harnett MM (2009). Immunomodulatory activity and therapeutic potential of the filarial nematode secreted product, ES-62. Adv Exp Med Biol.

[CR87] Harnett W, Worms MJ, Kapil A, Grainger M, Parkhouse RM (1989). Origin, kinetics of circulation and fate in vivo of the major excretory-secretory product of Acanthocheilonema viteae. Parasitology..

[CR88] Harnett W, Deehan MR, Houston KM, Harnett MM (1999). Immunomodulatory properties of a phosphorylcholine-containing secreted filarial glycoprotein. Parasite Immunol.

[CR89] Harnett W, McInnes IB, Harnett MM (2004). ES-62, a filarial nematode-derived immunomodulator with anti-inflammatory potential. Immunol Lett.

[CR90] Hartmann S, Adam R, Marti T, Kirsten C, Seidinger S, Lucius R (1997). A 41-kDa antigen of the rodent filaria Acanthocheilonema viteae with homologies to tropomyosin induces host-protective immune responses. Parasitol Res.

[CR91] Hartmann S, Kyewski B, Sonnenburg B, Lucius R (1997). A filarial cysteine protease inhibitor down-regulates T cell proliferation and enhances interleukin-10 production. Eur J Immunol.

[CR92] Hartmann W, Linnemann LC, Reitz M, Specht S, Voehringer D, Breloer M (2018). Basophils are dispensable for the control of a filarial infection. Immunohorizons..

[CR93] Hartmann W, Brunn ML, Stetter N, Gagliani N, Muscate F, Stanelle-Bertram S, Gabriel G, Breloer M (2019). Helminth infections suppress the efficacy of vaccination against seasonal influenza. Cell Rep.

[CR94] Haslam SM, Houston KM, Harnett W, Reason AJ, Morris HR, Dell A (1999). Structural studies of N-glycans of filarial parasites. Conservation of phosphorylcholine-substituted glycans among species and discovery of novel chito-oligomers. J Biol Chem.

[CR95] Hawking F (1962). The role of the spleen in controlling the number of microfilariae (Dirofilaria immitis, D. repens, Litomosoides carinii and Dipetalonema witei) in the blood. Ann Trop Med Parasitol.

[CR96] Hawking F, Burroughs AM (1946). Transmission of Litomosoides carinii to mice and hamsters. Nature.

[CR97] Hawking F, Clark JB (1967). The periodicity of microfilariae. 13. Movements of Dipetalonema witei microfilariae in the lungs. Trans R Soc Trop Med Hyg.

[CR98] Hawking F, Sewell P, Davey PD (1947). The maintenance of a filarial infection (Litomosoides carinii) in the laboratory. Trans R Soc Trop Med Hyg.

[CR99] Hawking F, Sewell P, Thurston JP (1950). The mode of action of hetrazan on filarial worms. Br J Pharmacol Chemother.

[CR100] Hawryluk NA (2020). Macrofilaricides: an unmet medical need for filarial diseases. ACS Infect Dis.

[CR101] Hewitt RI, White E, Wallace WS, Stewart HW, Kushner S, Subbarow Y (1947). Experimental chemotherapy of filariasis; effect of piperazine derivatives against naturally acquired filarial infections in cotton rats and dogs. J Lab Clin Med.

[CR102] Hillier SD, Booth M, Muhangi L, Nkurunziza P, Khihembo M, Kakande M, Sewankambo M, Kizindo R, Kizza M, Muwanga M, Elliott AM (2008). Plasmodium falciparum and helminth coinfection in a semi urban population of pregnant women in Uganda. J Infect Dis.

[CR103] Hoerauf A (2009). Mansonella perstans--the importance of an endosymbiont. N Engl J Med.

[CR104] Hoerauf A, Brattig N (2002). Resistance and susceptibility in human onchocerciasis--beyond Th1 vs. Th2. Trends Parasitol.

[CR105] Hoerauf A, Nissen-Pähle K, Schmetz C, Henkle-Dührsen K, Blaxter ML, Büttner DW, Gallin MY, Al-Qaoud KM, Lucius R, Fleischer B (1999). Tetracycline therapy targets intracellular bacteria in the filarial nematode Litomosoides sigmodontis and results in filarial infertility. J Clin Invest.

[CR106] Hoerauf A, Volkmann L, Hamelmann C, Adjei O, Autenrieth IB, Fleischer B, Büttner DW (2000). Endosymbiotic bacteria in worms as targets for a novel chemotherapy in filariasis. Lancet..

[CR107] Hoerauf A, Mand S, Adjei O, Fleischer B, Büttner DW (2001). Depletion of wolbachia endobacteria in Onchocerca volvulus by doxycycline and microfilaridermia after ivermectin treatment. Lancet..

[CR108] Hoerauf A, Adjei O, Büttner DW (2002). Antibiotics for the treatment of onchocerciasis and other filarial infections. Curr Opin Investig Drugs.

[CR109] Hoerauf A, Mand S, Fischer K, Kruppa T, Marfo-Debrekyei Y, Debrah AY, Pfarr KM, Adjei O, Büttner DW (2003). Doxycycline as a novel strategy against bancroftian filariasis-depletion of Wolbachia endosymbionts from Wuchereria bancrofti and stop of microfilaria production. Med Microbiol Immunol.

[CR110] Hoerauf A, Mand S, Volkmann L, Büttner M, Marfo-Debrekyei Y, Taylor M, Adjei O, Büttner DW (2003). Doxycycline in the treatment of human onchocerciasis: kinetics of Wolbachia endobacteria reduction and of inhibition of embryogenesis in female Onchocerca worms. Microbes Infect.

[CR111] Hoffmann W, Petit G, Schulz-Key H, Taylor D, Bain O, Le Goff L (2000). Litomosoides sigmodontis in mice: reappraisal of an old model for filarial research. Parasitol Today.

[CR112] Holdstock RP (1974). Proceedings: Mastomys natalensis as a host for Dipetalonema viteae. Trans R Soc Trop Med Hyg.

[CR113] Hong WD, Benayoud F, Nixon GL, Ford L, Johnston KL, Clare RH, Cassidy A, DAN C, Siu A, Shiotani M, Webborn PJH, Kavanagh S, Aljayyoussi G, Murphy E, Steven A, Archer J, Struever D, Frohberger SJ, Ehrens A, Hübner MP, Hoerauf A, Roberts AP, ATM H, Tate EW, Serwa RA, Leung SC, Qie L, Berry NG, Gusovsky F, Hemingway J, Turner JD, Taylor MJ, Ward SA, O’Neill PM (2019). AWZ1066S, a highly specific anti-Wolbachia drug candidate for a short-course treatment of filariasis. Proc Natl Acad Sci U S A.

[CR114] https://dndi.org/diseases/filaria-river-blindness/projects-achievements/, (2020) accessed 19^th^ October 2020

[CR115] Hübner MP, Martin C, Specht S, Koschel M, Dubben B, Frohberger SJ, Ehrens A, Fendler M, Struever D, Mitre E, Vallarino-Lhermitte N, Gokool S, Lustigman S, Schneider M, Townson S, Hoerauf A, Scandale I (2020) Oxfendazole mediates macrofilaricidal efficacy against the filarial nematode Litomosoides sigmodontis in vivo and inhibits Onchocerca spec. motility in vitro. PLoS Negl Trop Dis 14(7):e0008427 10.1371/journal.pntd.0008427 PMC7365463 32628671

[CR116] Hübner MP, Torrero MN, McCall JW, Mitre E (2009). Litomosoides sigmodontis: a simple method to infect mice with L3 larvae obtained from the pleural space of recently infected jirds (Meriones unguiculatus). Exp Parasitol.

[CR117] Hübner MP, Killoran KE, Rajnik M, Wilson S, Yim KC, Torrero MN, Morris CP, Nikonenko B, Blanco JC, Hemming VG, Mitre E (2012). Chronic helminth infection does not exacerbate Mycobacterium tuberculosis infection. PLoS Negl Trop Dis.

[CR118] Hübner MP, Shi Y, Torrero MN, Mueller E, Larson D, Soloviova K, Gondorf F, Hoerauf A, Killoran KE, Stocker JT, Davies SJ, Tarbell KV, Mitre E (2012). Helminth protection against autoimmune diabetes in nonobese diabetic mice is independent of a type 2 immune shift and requires TGF-β. J Immunol.

[CR119] Hübner MP, Ehrens A, Koschel M, Dubben B, Lenz F, Frohberger SJ, Specht S, Quirynen L, Lachau-Durand S, Tekle F, Baeten B, Engelen M, Mackenzie CD, Hoerauf A (2019). Macrofilaricidal efficacy of single and repeated oral and subcutaneous doses of flubendazole in Litomosoides sigmodontis infected jirds. PLoS Negl Trop Dis.

[CR120] Hübner MP, Koschel M, Struever D, Nikolov V, Frohberger SJ, Ehrens A, Fendler M, Johannes I, von Geldern TW, Marsh K, Turner JD, Taylor MJ, Ward SA, Pfarr K, Kempf DJ, Hoerauf A (2019). In vivo kinetics of Wolbachia depletion by ABBV-4083 in L. sigmodontis adult worms and microfilariae. PLoS Negl Trop Dis.

[CR121] Hübner MP, Martin C, Specht S, Koschel M, Dubben B, Frohberger SJ, Ehrens A, Fendler M, Struever D, Mitre E, Vallarino-Lhermitte N, Gokool S, Lustigman S, Schneider M, Townson S, Hoerauf A, Scandale I (2020). Oxfendazole mediates macrofilaricidal efficacy against the filarial nematode Litomosoides sigmodontis in vivo and inhibits Onchocerca spec. motility in vitro. PLoS Negl Trop Dis.

[CR122] Jacobs RT, Lunde CS, Freund YR, Hernandez V, Li X, Xia Y, Carter DS, Berry PW, Halladay J, Rock F, Stefanakis R, Easom E, Plattner JJ, Ford L, Johnston KL, Cook DAN, Clare R, Cassidy A, Myhill L, Tyrer H, Gamble J, Guimaraes AF, Steven A, Lenz F, Ehrens A, Frohberger SJ, Koschel M, Hoerauf A, Hübner MP, McNamara CW, Bakowski MA, Turner JD, Taylor MJ, Ward SA (2019). Boron-pleuromutilins as anti- Wolbachia agents with potential for treatment of onchocerciasis and lymphatic filariasis. J Med Chem.

[CR123] Jenkins SJ, Ruckerl D, Cook PC, Jones LH, Finkelman FD, van Rooijen N, MacDonald AS, Allen JE (2011). Local macrophage proliferation, rather than recruitment from the blood, is a signature of TH2 inflammation. Science.

[CR124] Johnson MH, Orihel TC, Beaver PC (1974). Dipetalonema viteae in the experimentally infected jird, Meriones unguiculatus. I. Insemination, development from egg to microfilaria, reinsemination, and longevity of mated and unmated worms. J Parasitol.

[CR125] Kabagenyi J, Natukunda A, Nassuuna J, Sanya RE, Nampijja M, Webb EL, Elliott AM, Nkurunungi G (2020). Urban-rural differences in immune responses to mycobacterial and tetanus vaccine antigens in a tropical setting: a role for helminths?. Parasitol Int.

[CR126] Kamtchum Tatuene JGFR, Nkoa T, Tchateng Mbougua JB, Nana Djeunga HC, Bopda J, Kamgno J (2014). Epidemiology of Loa loa and Mansonella perstans filariasis in the Akonolinga health district, centre region, Cameroon. Health Sci Dis.

[CR127] Karadjian G, Berrebi D, Dogna N, Vallarino-Lhermitte N, Bain O, Landau I, Martin C (2014). Co-infection restrains Litomosoides sigmodontis filarial load and plasmodial P. yoelii but not P. chabaudi parasitaemia in mice. Parasite.

[CR128] Karadjian G, Fercoq F, Pionnier N, Vallarino-Lhermitte N, Lefoulon E, Nieguitsila A, Specht S, Carlin LM, Martin C (2017). Migratory phase of Litomosoides sigmodontis filarial infective larvae is associated with pathology and transient increase of S100A9 expressing neutrophils in the lung. PLoS Negl Trop Dis.

[CR129] Katawa G, Layland LE, Debrah AY, von Horn C, Batsa L, Kwarteng A, Arriens SW, Taylor D, Specht S, Hoerauf A, Adjobimey T (2015). Hyperreactive onchocerciasis is characterized by a combination of Th17-Th2 immune responses and reduced regulatory T cells. PLoS Negl Trop Dis.

[CR130] Kelly-Hope LA, Bockarie MJ, Molyneux DH (2012). Loa loa ecology in central Africa: role of the Congo River system. PLoS Negl Trop Dis.

[CR131] Kershaw WE (1953). The early migration-rate of the infective larva of Litomosoides carinii in the cotton rat. Ann Trop Med Parasitol.

[CR132] Kershaw WE, Plackett RL (1948). Observations on Litomosoides carinii (Travassos, 1919) Chandler, 1931; the development of the first-stage larva. Ann Trop Med Parasitol.

[CR133] Kilarski WW, Martin C, Pisano M, Bain O, Babayan SA, Swartz MA (2019). Inherent biomechanical traits enable infective filariae to disseminate through collecting lymphatic vessels. Nat Commun.

[CR134] Klarmann U, Debrah AY, Mand S, Batsa L (2012). Shortening the timeframe and dosage of antiwolbachia therapy: doxycycline alone versus doxycycline plus rifampicin in their efficacy against lymphatic filariasis; a randomized, doubleblind, placebo-controlled trial. Am J Trop Med Hyg.

[CR135] Klarmann-Schulz U, Specht S, Debrah AY, Batsa L (2017). Comparison of doxycycline, minocycline, doxycycline plus albendazole and albendazole alone in their efficacy against onchocerciasis in a randomized, open-label, pilot trial. PLoS Negl Trop Dis.

[CR136] Knipper JA, Ivens A, Taylor MD (2019). Helminth-induced Th2 cell dysfunction is distinct from exhaustion and is maintained in the absence of antigen. PLoS Negl Trop Dis.

[CR137] Kolbaum J, Tartz S, Hartmann W, Helm S, Nagel A, Heussler V, Sebo P, Fleischer B, Jacobs T, Breloer M (2012). Nematode-induced interference with the anti-Plasmodium CD8+ T-cell response can be overcome by optimizing antigen administration. Eur J Immunol.

[CR138] Koudou BG, de Souza DK, Biritwum NK, Bougma R, Aboulaye M, Elhassan E, Bush S, Molyneux DH (2018). Elimination of lymphatic filariasis in west African urban areas: is implementation of mass drug administration necessary?. Lancet Infect Dis.

[CR139] Kozek, Wieslaw J., and Ramakrishna U. Rao. “The discovery of Wolbachia in arthropods and nematodes–a historical perspective.” Wolbachia: a bug's life in another bug. Vol. 5. Karger Publishers, 2007. 1-14.

[CR140] Krepkogorskaja: Rev. Microbiol. Epidemiol. et Parasitol. 1933

[CR141] Kroidl I, Saathoff E, Maganga L, Makunde WH, Hoerauf A, Geldmacher C, Clowes P, Maboko L, Hoelscher M (2016). Effect of Wuchereria bancrofti infection on HIV incidence in southwest Tanzania: a prospective cohort study. Lancet.

[CR142] Kulke D, Townson S, Bloemker D, Frohberger S, Specht S, Scandale I (2017). Comparison of the in vitro susceptibility to emodepside of microfilariae, third stage larvae and adult worms of related filarial nematodes. Am J Trop Med Hyg.

[CR143] Lachau-Durand S, Lammens L, van der Leede BJ, Van Gompel J, Bailey G, Engelen M, Lampo A (2019). Preclinical toxicity and pharmacokinetics of a new orally bioavailable flubendazole formulation and the impact for clinical trials and risk/benefit to patients. PLoS Negl Trop Dis.

[CR144] Laing R, Gillan V, Devaney E (2017). Ivermectin–old drug, new tricks?. Trends Parasitol.

[CR145] Langdon K, Phie J, Thapa CB, Biros E, Loukas A, Haleagrahara N (2019). Helminth-based therapies for rheumatoid arthritis: a systematic review and meta-analysis. Int Immunopharmacol.

[CR146] Larson D, Hübner MP, Torrero MN, Morris CP, Brankin A, Swierczewski BE, Davies SJ, Vonakis BM, Mitre E (2012). Chronic helminth infection reduces basophil responsiveness in an IL-10-dependent manner. J Immunol.

[CR147] Lawrence RA, Allen JE, Osborne J, Maizels RM (1994). Adult and microfilarial stages of the filarial parasite Brugia malayi stimulate contrasting cytokine and Ig isotype responses in BALB/c mice. J Immunol.

[CR148] Layland LE, Ajendra J, Ritter M, Wiszniewsky A, Hoerauf A, Hübner MP (2015). Development of patent Litomosoides sigmodontis infections in semi-susceptible C57BL/6 mice in the absence of adaptive immune responses. Parasit Vectors.

[CR149] Le Goff L, Loke P, Ali HF, Taylor DW, Allen JE (2000). Interleukin-5 is essential for vaccine-mediated immunity but not innate resistance to a filarial parasite. Infect Immun.

[CR150] Lucius R, Textor G, Kern A, Kirsten C (1991). Acanthocheilonema viteae: vaccination of jirds with irradiation-attenuated stage-3 larvae and with exported larval antigens. Exp Parasitol.

[CR151] Lukiana T, Mandina M, Situakibanza NH, Mbula MM, Lepira BF, Odio WT, Kamgno J, Boussinesq M (2006). A possible case of spontaneous Loa loa encephalopathy associated with a glomerulopathy. Filaria J.

[CR152] Lumb FE, Doonan J, Corbet M, Pineda MA, M Harnett M, Harnett W. (2020). Development of Acanthocheilonema viteae in Meriones shawi: absence of microfilariae and production of active ES-62. Parasite Immunol.

[CR153] Macdonald EM, Scott JA (1958). The persistence of acquired immunity to the filarial worm of the cotton rat. Am J Trop Med Hyg.

[CR154] Macfarlane CL, Budhathoki SS, Johnson S, Richardson M, Garner P (2019). Albendazole alone or in combination with microfilaricidal drugs for lymphatic filariasis. Cochrane Database Syst Rev.

[CR155] Maizels RM, Denham DA (1992). Diethylcarbamazine (DEC): immunopharmacological interactions of an anti-filarial drug. Parasitology.

[CR156] Maizels RM, Smits HH, McSorley HJ (2018). Modulation of host immunity by helminths: the expanding repertoire of parasite effector molecules. Immunity.

[CR157] Marcoullis G, Gräsbeck R (1976). Preliminary identification and characterization of antigen extracts from Onchocerca volvulus. Tropenmed Parasitol.

[CR158] Martin C (2014). Odile Bain (April 28, 1939–October 16, 2012): A life dedicated to systematics and biology of filariae. PLoS Negl Trop Dis.

[CR159] Martin C, Al-Qaoud KM, Ungeheuer MN, Paehle K, Vuong PN, Bain O, Fleischer B, Hoerauf A (2000). IL-5 is essential for vaccine-induced protection and for resolution of primary infection in murine filariasis. Med Microbiol Immunol.

[CR160] Martin C, Le Goff L, Ungeheuer MN, Vuong PN, Bain O (2000). Drastic reduction of a filarial infection in eosinophilic interleukin-5 transgenic mice. Infect Immun.

[CR161] Martin C, Saeftel M, Vuong PN, Babayan S, Fischer K, Bain O, Hoerauf A (2001). B-cell deficiency suppresses vaccine-induced protection against murine filariasis but does not increase the recovery rate for primary infection. Infect Immun.

[CR162] Mauricio S, Lindsay JR, Frank OR (2017). Progress toward elimination of onchocerciasis in the Americas. J Int Health.

[CR163] McCall JW (1976). A simple method for collecting infective larvae of Litomosoides carinii. J Parasitol.

[CR164] McClure WR, Cech CL (1978). On the mechanism of rifampicin inhibition of RNA synthesis. J Biol Chem.

[CR165] McLaren DJ, Worms MJ, Laurence BR, Simpson MG (1975). Micro-organisms in filarial larvae (Nematoda). Trans R Soc Trop Med Hyg.

[CR166] Mehta K, Sindhu RK, Subrahmanyam D, Nelson DS (1980). IgE-dependent adherence and cytotoxicity of rat spleen and peritoneal cells to Litomosoides carinii microfilariae. Clin Exp Immunol.

[CR167] Mehta K, Sindhu RK, Subrahmanyam D, Hopper K, Nelson DS (1982). IgE-dependent cellular adhesion and cytotoxicity to Litomosoides carinii microfilariae--nature of effector cells. Clin Exp Immunol.

[CR168] Mhimbira F, Hella J, Said K, Kamwela L, Sasamalo M, Maroa T, Chiryamkubi M, Mhalu G, Schindler C, Reither K, Knopp S, Utzinger J, Gagneux S, Fenner L (2017). Prevalence and clinical relevance of helminth co-infections among tuberculosis patients in urban Tanzania. PLoS Negl Trop Dis.

[CR169] Midha A, Janek K, Niewienda A, Henklein P, Guenther S, Serra DO, Schlosser J, Hengge R, Hartmann S (2018). The intestinal roundworm Ascaris suum releases antimicrobial factors which interfere with bacterial growth and biofilm formation. Front Cell Infect Microbiol.

[CR170] Milton P (2020). Moxidectin: an oral treatment for human onchocerciasis. Expert Rev Anti-Infect Ther.

[CR171] Morris CP, Evans H, Larsen SE, Mitre E (2013). A comprehensive, model-based review of vaccine and repeat infection trials for filariasis. Clin Microbiol Rev.

[CR172] Muhangi L, Woodburn P, Omara M, Omoding N, Kizito D, Mpairwe H, Nabulime J, Ameke C, Morison LA, Elliott AM (2007). Associations between mild-to-moderate anaemia in pregnancy and helminth, malaria and HIV infection in Entebbe, Uganda. Trans R Soc Trop Med Hyg.

[CR173] Muhsin M, Ajendra J, Gentil K, Berbudi A, Neumann AL, Klaas L, Schmidt KE, Hoerauf A, Hübner MP (2018). IL-6 is required for protective immune responses against early filarial infection. Int J Parasitol.

[CR174] Nakamura M, Nogami S, Hayashi Y, Shibuya T, Tanaka H (1984). A mass dissection method for collecting infective larvae of Litomosoides carinii from mites. Jpn J Exp Med.

[CR175] Neilson JT (1978). Alteration of amicrofilaremia in Dipetalonema viteae infected hamsters with immunosuppressive drugs. Acta Trop.

[CR176] Neilson JT, Forrester DJ (1975). Dipetalonema viteae: primary, secondary and tertiary infections in hamsters. Exp Parasitol.

[CR177] Njim T, Ngum JM, Aminde LN (2015). Cutaneous onchocerciasis in Dumbu, a pastoral area in the North-West region of Cameroon: diagnostic challenge and socio-economic implications. Pan Afr Med J.

[CR178] Njouendou AJ, Ritter M, Ndongmo WPC, Kien CA, Narcisse GTV, Fombad FF, Tayong DB, Pfarr K, Layland LE, Hoerauf A, Wanji S (2017). Successful long-term maintenance of Mansonella perstans in an in vitro culture system. Parasit Vectors.

[CR179] Njouendou AJ, Fombad FF, O'Neill M, Zofou D, Nutting C, Ndongmo PC, Kengne-Ouafo AJ, Geary TG, Mackenzie CD, Wanji S (2018). Heterogeneity in the in vitro susceptibility of Loa loa microfilariae to drugs commonly used in parasitological infections. Parasit Vectors.

[CR180] Njouendou AJ, Kien CA, Esum ME, Ritter M, Chounna Ndongmo WP, Fombad FF, Gandjui NVT, Njiokou F, Enyong P, Pfarr K, Turner J, Layland LE, Hoerauf A, Wanji S (2019). In vitro maintenance of Mansonella perstans microfilariae and its relevance for drug screening. Exp Parasitol.

[CR181] Opoku NO, Bakajika DK, Kanza EM, Howard H, Mambandu GL, Nyathirombo A, ... & Kataliko K (2018). Single dose moxidectin versus ivermectin for Onchocerca volvulus infection in Ghana, Liberia, and the Democratic Republic of the Congo: a randomised, controlled, double-blind phase 3 trial. The Lancet, 392(10154), 1207-1216. 10.1016/S0140-6736(17)32844-1 PMC6172290 29361335

[CR182] Pacheco G (1970). Infection and superinfection of jirds (Meriones unguiculatus) and hamsters (Mesocricetus auratus) with Dipetalonema viteae. J Parasitol.

[CR183] Padgett JJ, Jacobsen KH (2008). Loiasis: African eye worm. Trans R Soc Trop Med Hyg.

[CR184] Patra, BB, Basu UP. (1970) Parasitic development of Litomosoides carinii in some rodents. Proc Indian Sci Congress Assoc. 57. (III)

[CR185] Patton JB, Bennuru S, Eberhard ML, Hess JA, Torigian A, Lustigman S, Nutman TB, Abraham D (2018). Development of Onchocerca volvulus in humanized NSG mice and detection of parasite biomarkers in urine and serum. PLoS Negl Trop Dis.

[CR186] Petit G, Diagne M, Maréchal P, Owen D, Taylor D, Bain O (1992). Maturation of the filaria Litomosoides sigmodontis in BALB/c mice; comparative susceptibility of nine other inbred strains. Ann Parasitol Hum Comp.

[CR187] Pfaff AW, Schulz-Key H, Soboslay PT, Taylor DW, MacLennan K, Hoffmann WH (2002). Litomosoides sigmodontis cystatin acts as an immunomodulator during experimental filariasis. Int J Parasitol.

[CR188] Pfarr KM, Fischer K, Hoerauf A (2003). Involvement of Toll-like receptor 4 in the embryogenesis of the rodent filaria Litomosoides sigmodontis. Med Microbiol Immunol.

[CR189] Pineda MA, Al-Riyami L, Harnett W, Harnett MM (2014). Lessons from helminth infections: ES-62 highlights new interventional approaches in rheumatoid arthritis. Clin Exp Immunol.

[CR190] Pineda MA, Lumb F, Harnett MM, Harnett W (2014). ES-62, a therapeutic anti-inflammatory agent evolved by the filarial nematode Acanthocheilonema viteae. Mol Biochem Parasitol.

[CR191] Pineda MA, Eason RJ, Harnett MM, Harnett W (2015). From the worm to the pill, the parasitic worm product ES-62 raises new horizons in the treatment of rheumatoid arthritis. Lupus..

[CR192] Pionnier N, Brotin E, Karadjian G, Hemon P, Gaudin-Nomé F, Vallarino-Lhermitte N, Nieguitsila A, Fercoq F, Aknin ML, Marin-Esteban V, Chollet-Martin S, Schlecht-Louf G, Bachelerie F, Martin C (2016) Neutropenic Mice Provide Insight into the Role of Skin-Infiltrating Neutrophils in the Host Protective Immunity against Filarial Infective Larvae. PLoS Negl Trop Dis 10(4):e0004605 10.1371/journal.pntd.0004605 PMC4844152 27111140

[CR193] Pionnier NP, Sjoberg H, Chunda VC, Fombad FF, Chounna PW, Njouendou AJ, Metuge HM, Ndzeshang BL, Gandjui NV, Akumtoh DN, Tayong DB, Taylor MJ, Wanji S, Turner JD (2019). Mouse models of Loa loa. Nat Commun.

[CR194] Pringle G, King DF (1968). Some developments in techniques for the study of the rodent filarial parasite Litomosoides carinii. I. A preliminary comparison of the host effciency of the multimammate rat, Praomys (Mastomys) natalensis, with that of the cotton rat, Sigmodon hispidus. Ann Trop Med Parasitol.

[CR195] Quintana JF, Kumar S, Ivens A, Chow FWN, Hoy AM, Fulton A, Dickinson P, Martin C, Taylor M, Babayan SA, Buck AH (2019). Comparative analysis of small RNAs released by the filarial nematode Litomosoides sigmodontis in vitro and in vivo. PLoS Negl Trop Dis.

[CR196] Ramaiah KD, Ottesen EA (2014). Progress and impact of 13 years of the global programme to eliminate lymphatic filariasis on reducing the burden of filarial disease. PLoS Negl Trop Dis.

[CR197] Ramakrishnan SP, Singh D, Bhatnagar VN, Raghavan NG (1961). Infection of the albino rat with the filarial parasite. Litomosoides carinii, of cotton rats. Indian J Malariol.

[CR198] Rao UR, Chandrashekar R, Subrahmanyam D (1987). Effect of ivermectin on serum dependent cellular interactions to Dipetalonema viteae microfilariae. Trop Med Parasitol.

[CR199] Rao UR, Chandrashekar R, Subrahmanyam D (1990). Effect of ivermectin on filariae of Mastomys natalensis. Parasitol Res.

[CR200] Rausch S, Midha A, Kuhring M, Affinass N, Radonic A, Kühl AA, Bleich A, Renard BY, Hartmann S (2018). Parasitic nematodes exert antimicrobial activity and benefit from microbiota-driven support for host immune regulation. Front Immunol.

[CR201] Rebollo MP, Bockarie MJ (2017). Can lymphatic filariasis be eliminated by 2020?. Trends Parasitol.

[CR202] Reddy AB, Rao UR, Chandrashekar R, Shrivastava R, Subrahmanyam D (1983). Comparative efficacy of some benzimidazoles and amoscanate (Go.9333) against experimental filarial infections. Tropenmed Parasitol.

[CR203] Richard-Lenoble D, Chandenier J, Gaxotte P (2003). Ivermectin and filariasis. Fundam Clin Pharmacol.

[CR204] Ritter M, Tamadaho RS, Feid J, Vogel W, Wiszniewsky K, Perner S, Hoerauf A, Layland LE (2017). IL-4/5 signalling plays an important role during Litomosoides sigmodontis infection, influencing both immune system regulation and tissue pathology in the thoracic cavity. Int J Parasitol.

[CR205] Ritter M, Krupp V, Wiszniewsky K, Wiszniewsky A, Katawa G, Tamadaho RSE, Hoerauf A, Layland LE (2018). Absence of IL-17A in Litomosoides sigmodontis-infected mice influences worm development and drives elevated filarial-specific IFN-γ. Parasitol Res.

[CR206] Ritter M, Ndongmo WPC, Njouendou AJ, Nghochuzie NN, Nchang LC, Tayong DB, Arndts K, Nausch N, Jacobsen M, Wanji S, Layland LE, Hoerauf A (2018). Mansonella perstans microfilaremic individuals are characterized by enhanced type 2 helper T and regulatory T and B cell subsets and dampened systemic innate and adaptive immune responses. PLoS Negl Trop Dis.

[CR207] Ritter M, Osei-Mensah J, Debrah LB, Kwarteng A, Mubarik Y, Debrah AY, Pfarr K, Hoerauf A, Layland LE (2019). Wuchereria bancrofti-infected individuals harbor distinct IL-10-producing regulatory B and T cell subsets which are affected by anti-filarial treatment. PLoS Negl Trop Dis.

[CR208] Rodrigo MB, Schulz S, Krupp V, Ritter M, Wiszniewsky K, Arndts K, Tamadaho RS, Endl E, Hoerauf A, Layland LE (2016). Patency of Litomosoides sigmodontis infection depends on Toll-like receptor 4 whereas Toll-like receptor 2 signalling influences filarial-specific CD4(+) T-cell responses. Immunology.

[CR209] Saeftel M, Arndt M, Specht S, Volkmann L, Hoerauf A (2003). Synergism of gamma interferon and interleukin-5 in the control of murine filariasis. Infect Immun.

[CR210] Saint André AV, Blackwell NM, Hall LR, Hoerauf A, Brattig NW, Volkmann L, Taylor MJ, Ford L, Hise AG, Lass JH, Diaconu E, Pearlman E (2002). The role of endosymbiotic Wolbachia bacteria in the pathogenesis of river blindness. Science..

[CR211] Sänger I, Lämmler G (1979). On Dipetalonema viteae infection of Mastomys natalensis. Tropenmed Parasitol.

[CR212] Santiago HC, Nutman TB (2016). Human helminths and allergic disease: the hygiene hypothesis and beyond. Am J Trop Med Hyg.

[CR213] Santiago-Stevenson D, Oliver-Gonzalez J, HEWITT R (1947). Treatment of filariasis bancrofti with 1-diethylcarbamyl-4-methylpiperazine hydrochloride (hetrazan). J Am Med Assoc.

[CR214] Schaberle TF, Schmitz A, Zocher G, Schiefer A (2015). Insights into structure-activity relationships of bacterial RNA polymerase inhibiting corallopyronin derivatives. J Nat Prod.

[CR215] Schardein JL, Lucas JA, Dickerson CW (1968). Ultrastructural changes in Litomosoides carinii microfilariae in gerbils treated with diethylcarbamazine. J Parasitol.

[CR216] Schares G, Hofmann B, Zahner H (1994). Antifilarial activity of macrocyclic lactones: comparative studies with ivermectin, doramectin, milbemycin A4 oxime, and moxidectin in Litomosoides carinii, Acanthocheilonema viteae, Brugia malayi, and B. pahangi infection of Mastomys coucha. Trop Med Parasitol.

[CR217] Schiefer A, Schmitz A, Schäberle TF, Specht S, Lämmer C, Johnston KL, Vassylyev DG, König GM, Hoerauf A, Pfarr K (2012). Corallopyronin A specifically targets and depletes essential obligate Wolbachia endobacteria from filarial nematodes in vivo. J Infect Dis.

[CR218] Schiefer A, Hübner MP, Krome A, Lämmer C, Ehrens A, Aden T, Koschel M, Neufeld H, Chaverra-Muñoz L, Jansen R, Kehraus S, König GM, Pogorevc D, Müller R, Stadler M, Hüttel S, Hesterkamp T, Wagner K, Pfarr K, Hoerauf A (2020). Corallopyronin A for short-course anti-wolbachial, macrofilaricidal treatment of filarial infections. PLoS Negl Trop Dis.

[CR219] Schneider CR, Blair LS, Schardein JL, Boche LK, Thompson PE (1968). Comparison of early Litomosoides carinii infections in cotton rats and gerbils. J Parasitol.

[CR220] Scott JA, Macdonald EM (1958). Immunity to challenging infections of Litomosoides carinii produced by transfer of developing worms. J Parasitol.

[CR221] Scott JA, Stembridge VA, Sisley NM (1947). A method for providing a constant supply of tropical rat mites, Liponyssus bacoti, infected with the cotton rat filaria, Litosomoides carinii. J Parasitol.

[CR222] Simonsen PE, Onapa AW, Asio SM (2011). Mansonella perstans filariasis in Africa. Acta Trop.

[CR223] Singh DP, Rathore S, Misra S, Chatterjee RK, Ghatak S, Sen AB (1985). Studies on the causation of adverse reactions in microfilaraemic host following diethylcarbamazine therapy (Dipetalonema viteae in Mastomys natalensis). Trop Med Parasitol.

[CR224] Sironi M, Bandi C, Sacchi L, Di Sacco B, Damiani G, Genchi C (1995). Molecular evidence for a close relative of the arthropod endosymbiont Wolbachia in a filarial worm. Mol Biochem Parasitol.

[CR225] Specht S, Saeftel M, Arndt M, Endl E, Dubben B, Lee NA, Lee JJ, Hoerauf A (2006). Lack of eosinophil peroxidase or major basic protein impairs defense against murine filarial infection. Infect Immun.

[CR226] Specht S, Mand S, Marfo-Debrekyei Y, Debrah AY, Konadu P, Adjei O, Buttner DW, Hoerauf A (2008). Efficacy of 2- and 4-week rifampicin treatment on the Wolbachia of Onchocerca volvulus. Parasitol Res.

[CR227] Specht S, Ruiz DF, Dubben B, Deininger S, Hoerauf A (2010). Filaria-induced IL-10 suppresses murine cerebral malaria. Microbes Infect.

[CR228] Specht S, Pfarr KM, Arriens S, Hübner MP (2018). Combinations of registered drugs reduce treatment times required to deplete Wolbachia in the Litomosoides sigmodontis mouse model. PLoS Negl Trop Dis.

[CR229] Stensgaard AS, Vounatsou P, Onapa AW, Utzinger J, Pedersen EM, Kristensen TK, Simonsen PE (2016). Ecological drivers of Mansonella perstans infection in Uganda and patterns of co-endemicity with lymphatic filariasis and malaria. PLoS Negl Trop Dis.

[CR230] Storey N, Behnke JM, Wakelin D (1989). Acanthocheilonema viteae (Dipetalonema viteae) in mice: attempts to correct the low responder phenotype of the BALB/c host. Int J Parasitol.

[CR231] Supali T, Djuardi Y, Pfarr KM, Wibowo H, Taylor MJ, Hoerauf A, Houwing-Duistermaat JJ, Yazdanbakhsh M, Sartono E (2008). Doxycycline treatment of Brugia malayi-infected persons reduces microfilaremia and adverse reactions after diethylcarbamazine and albendazole treatment. Clin Infect Dis.

[CR232] Taylor MD, LeGoff L, Harris A, Malone E, Allen JE, Maizels RM (2005). Removal of regulatory T cell activity reverses hyporesponsiveness and leads to filarial parasite clearance in vivo. J Immunol.

[CR233] Taylor MD, Harris A, Nair MG, Maizels RM, Allen JE (2006). F4/80+ alternatively activated macrophages control CD4+ T cell hyporesponsiveness at sites peripheral to filarial infection. J Immunol.

[CR234] Taylor MD, Harris A, Babayan SA, Bain O, Culshaw A, Allen JE, Maizels RM (2007). CTLA-4 and CD4+ CD25+ regulatory T cells inhibit protective immunity to filarial parasites in vivo. J Immunol.

[CR235] Taylor MJ, Hoerauf A, Bockarie M (2010). Lymphatic filariasis and onchocerciasis. Lancet.

[CR236] Taylor MD, van der Werf N, Maizels RM (2012). T cells in helminth infection: the regulators and the regulated. Trends Immunol.

[CR237] Taylor MJ, von Geldern TW, Ford L, Hübner MP, Marsh K, Johnston KL, Sjoberg HT, Specht S, Pionnier N, Tyrer HE, Clare RH, Cook DAN, Murphy E, Steven A, Archer J, Bloemker D, Lenz F, Koschel M, Ehrens A, Metuge HM, Chunda VC, Ndongmo Chounna PW, Njouendou AJ, Fombad FF, Carr R, Morton HE, Aljayyoussi G, Hoerauf A, Wanji S, Kempf DJ, Turner JD, Ward SA (2019). Preclinical development of an oral anti-Wolbachia macrolide drug for the treatment of lymphatic filariasis and onchocerciasis. Sci Transl Med.

[CR238] Terry A, Tery RJ, Worms MJ (1961). Dipetalonema witei, filarial parasite of the jird, Meriones libycus. II. The reproductive system, gametogenesis and development of the microfilaria. J Parasitol.

[CR239] Tippawangkosol P, Choochote W, Riyong D, Jitpakdi A, Pitasawat B (2002). A simple technique for the in vitro cultivation of nocturnally subperiodic Brugia malayi infective larvae. Southeast Asian J Trop Med Public Health.

[CR240] Torrero MN, Hübner MP, Larson D, Karasuyama H, Mitre E (2010). Basophils amplify type 2 immune responses, but do not serve a protective role, during chronic infection of mice with the filarial nematode Litomosoides sigmodontis. J Immunol.

[CR241] Torrero MN, Morris CP, Mitre BK, Hübner MP, Fox EM, Karasuyama H, Mitre E (2013). Basophils help establish protective immunity induced by irradiated larval vaccination for filariasis. Vaccine.

[CR242] Traore MO, Sarr MD, Badji A, Bissan Y, Diawara L, Doumbia K, Goita SF, Konate L, Mounkoro K, Seck AF, Toe L, Toure S, Remme JH (2012). Proof-of-principle of onchocerciasis elimination with ivermectin treatment in endemic foci in Africa: final results of a study in Mali and Senegal. PLoS Negl Trop Dis.

[CR243] Travassos L (1919) Filaria carinii n. sp. Revista da Sociedade Brasileira de Ciência 3:189–190

[CR244] Trees AJ (1992). Onchocerca ochengi: Mimic, model or modulator of O. volvulus?. Parasitol Today.

[CR245] Tsalikis G (1993). The onchocerciasis control programme (OCP) in West Africa: a review of progress. Health Policy Plan.

[CR246] van der Werf N, Redpath SA, Azuma M, Yagita H, Taylor MD (2013). Th2 cell-intrinsic hypo-responsiveness determines susceptibility to helminth infection. PLoS Pathog.

[CR247] Vaz Z (1934). Ackertia gen. nov. for Litomosa burgosi De La Barrera, 1926, with notes on the synonymy and morphological variations of Litomosoides carinii (Travassos, 1919). Ann Trop Med Parasitol.

[CR248] Velásquez GE, Brooks MB, Coit JM, Pertinez H, Vargas Vásquez D, Sánchez Garavito E, Calderón RI, Jiménez J, Tintaya K, Peloquin CA, Osso E, Tierney DB, Seung KJ, Lecca L, Davies GR, Mitnick CD (2018). Efficacy and safety of high-dose rifampin in pulmonary tuberculosis. A randomized controlled trial. Am J Respir Crit Care Med.

[CR249] Veletzky L, Hergeth J, Stelzl DR, Mischlinger J, Manego RZ, Mombo-Ngoma G, McCall MBB, Adegnika AA, Agnandji ST, Metzger WG, Matsiegui PB, Lagler H, Mordmüller B, Budke C, Ramharter M (2020). Burden of disease in Gabon caused by loiasis: a cross-sectional survey. Lancet Infect Dis.

[CR250] Vijayan VK (2007). Tropical pulmonary eosinophilia: pathogenesis, diagnosis and management. Curr Opin Pulm Med.

[CR251] Vincent AL, Portaro JK, Ash LR (1975). A comparison of the body wall ultrastructure of Brugia pahangi with that of Brugia malayi. J Parasitol.

[CR252] Volkmann L, Saeftel M, Bain O, Fischer K, Fleischer B, Hoerauf A (2001). Interleukin-4 is essential for the control of microfilariae in murine infection with the filaria Litomosoides sigmodontis. Infect Immun.

[CR253] Volkmann L, Bain O, Saeftel M, Specht S, Fischer K, Brombacher F, Matthaei KI, Hoerauf A (2003). Murine filariasis: interleukin 4 and interleukin 5 lead to containment of different worm developmental stages. Med Microbiol Immunol.

[CR254] Volkmann L, Fischer K, Taylor M, Hoerauf A (2003). Antibiotic therapy in murine filariasis (Litomosoides sigmodontis): comparative effects of doxycycline and rifampicin on Wolbachia and filarial viability. Tropical Med Int Health.

[CR255] von Geldern TW, Morton HE, Clark RF, Brown BS, Johnston KL, Ford L, Specht S, Carr RA, Stolarik DF, Ma J, Rieser MJ, Struever D, Frohberger SJ, Koschel M, Ehrens A, Turner JD, Hübner MP, Hoerauf A, Taylor MJ, Ward SA, Marsh K, Kempf DJ (2019). Discovery of ABBV-4083, a novel analog of Tylosin A that has potent anti-Wolbachia and anti-filarial activity. PLoS Negl Trop Dis.

[CR256] Voronin D, Tricoche N, Jawahar S, Shlossman M, Bulman CA, Fischer C, Suderman MT, Sakanari JA, Lustigman S (2019). Development of a preliminary in vitro drug screening assay based on a newly established culturing system for pre-adult fifth-stage Onchocerca volvulus worms. PLoS Negl Trop Dis.

[CR257] Wanji S, Tayong DB, Layland LE, Datchoua Poutcheu FR, Ndongmo WP, Kengne-Ouafo JA, Ritter M, Amvongo-Adjia N, Fombad FF, Njeshi CN, Nkwescheu AS, Enyong PA, Hoerauf A (2016). Update on the distribution of Mansonella perstans in the southern part of Cameroon: influence of ecological factors and mass drug administration with ivermectin. Parasit Vectors.

[CR258] Wenk P (1967). Der Invasionsweg der metazyklischen Larven von Litomosoides carinii Chandler 1931 (Filariidae) [The invasion route of the metacyclical larvae of Litomosoides carinii Chandler 1931 (Filariidae)]. Z Parasitenkd.

[CR259] WHO (2015). Global programme to eliminate lymphatic filariasis: progress report, 2014. Wkly Epidemiol Rec.

[CR260] WHO (2018). Progress report on the elimination of human onchocerciasis, 2017-2018. Wkly Epidemiol Rec.

[CR261] WHO (2019). Progress in eliminating onchocerciasis in the WHO Region of the Americas: doxycycline treatment as an end-game strategy. Wkly Epidemiol Rep.

[CR262] WHO. Lymphatic filariasis. (2020) Available from: https://www.who.int/health-topics/lymphatic-filariasis#tab=tab_1. Accessed on 05. August 2020.

[CR263] WHO. Onchocerciasis. (2020) Available from: https://www.who.int/health-topics/onchocerciasis-(river-blindness)#tab=tab_1. Accessed on 05. August 2020.

[CR264] Williams RW (1948). Studies on the life cycle of Litomosoides carinii, filariid parasite of the cotton rat, Sigmodon hispidus litoralis. J Parasitol.

[CR265] Williams RW, Brown HW (1945). The development of Litomosoides carinii filariid parasite of the cotton rat in the tropical rat mite. Science..

[CR266] Wiszniewsky A, Ritter M, Krupp V, Schulz S, Arndts K, Weighardt H, Wanji S, Hoerauf A, Layland LE (2019). The central adaptor molecule TRIF influences L. sigmodontis worm development. Parasitol Res.

[CR267] Worms M, Terry R, Terry A (1961). Dipetalonema witei, filarial parasite of the jird, Meriones libycus. I. Maintenance in the Laboratory. J Parasitol.

[CR268] Zahner H, Schares G (1993). Experimental chemotherapy of filariasis: comparative evaluation of the efficacy of filaricidal compounds in Mastomys coucha infected with Litomosoides carinii, Acanthocheilonema viteae, Brugia malayi and B. pahangi. Acta Trop.

[CR269] Zahner H, Sänger I, Lämmler G, Müller HA (1987). Effect of ivermectin in Dipetalonema viteae and Litomosoides carinii infections of Mastomys natalensis. Trop Med Parasitol.

[CR270] Zahner H, Taubert A, Harder A, von Samson-Himmelstjerna G (2001). Effects of Bay 44-4400, a new cyclodepsipeptide, on developing stages of filariae (Acanthocheilonema viteae, Brugia malayi, Litomosoides sigmodontis) in the rodent Mastomys coucha. Acta Trop.

[CR271] Zofou D, Fombad FF, Gandjui NVT, Njouendou AJ, Kengne-Ouafo AJ, Chounna Ndongmo PW, Datchoua-Poutcheu FR, Enyong PA, Bita DT, Taylor MJ, Turner JD, Wanji S (2018). Evaluation of in vitro culture systems for the maintenance of microfilariae and infective larvae of Loa loa. Parasit Vectors.

[CR272] Zouré HG, Wanji S, Noma M, Amazigo UV, Diggle PJ, Tekle AH, Remme JH (2011). The geographic distribution of Loa loa in Africa: results of large-scale implementation of the Rapid Assessment Procedure for Loiasis (RAPLOA). PLoS Negl Trop Dis.

